# Female Budgerigars Attend Equally to Rhythmic Beat or Repetition, While Humans Prioritize Their Combination

**DOI:** 10.1111/nyas.70389

**Published:** 2026-09-22

**Authors:** Felix Haiduk, Daniel L. Bowling, Shahrzad Afroozeh, Jan Kopač, Mahin Azarian, Marisa Hoeschele

**Affiliations:** ^1^ Acoustics Research Institute Austrian Academy of Sciences Vienna Austria; ^2^ Department of General Psychology University of Padua Padua Italy; ^3^ Department of Behavioral and Cognitive Biology University of Vienna Vienna Austria; ^4^ Center for Computer Research in Music and Acoustics Stanford University Stanford California USA; ^5^ Department of Psychiatry and Behavioral Sciences Stanford University School of Medicine Stanford California USA

**Keywords:** animal cognition, beat, behavior, budgerigars, musicality, preference, rhythm

## Abstract

Rhythm is an essential aspect of human music, typically characterized by temporal regularity of sound events and repetition of rhythmic patterns. One key biological trait for human rhythmic behavior is the ability to entrain movement to sound events, an ability possibly related to vocal production learning. Parrots share both of these crucial traits with humans. Budgerigars in particular can entrain to a beat, and female budgerigars, like humans, prefer rhythmic sounds. What remains unclear is whether this attraction to rhythm is based on the same features that are important in humans. If so, this could suggest shared mechanisms and/or functions. Using a three‐choice place preference paradigm for both species, we found that female, but not male, budgerigars preferred rhythmic over arrhythmic stimuli, with equal preference for repetition and regularity. Humans preferred stimuli containing a regular beat, with a tendency to prioritize the combination of regularity and repetition. We suggest that female budgerigar preferences are driven by features of budgerigar warble song displayed in courtship contexts, while humans are driven by group chorusing behavior. We discuss how differential behavioral exploitation of shared biological traits may have had different evolutionary consequences and offer suggestions for how these can be tested.

## Graphical abstract

We investigated preferences of budgerigars and humans for rhythmic and arrhythmic sounds, specifically beat‐based regularity and repetition, using a place‐preference paradigm. We found that female budgerigars prefer regular and repetitive sounds equally, while humans prefer sounds containing a regular beat over repetition. We discuss the results with regard to different behavioral ecologies: budgerigar courtship and human chorusing, concluding that perceptual preferences depend on the exploitation of shared biological traits by behavior.

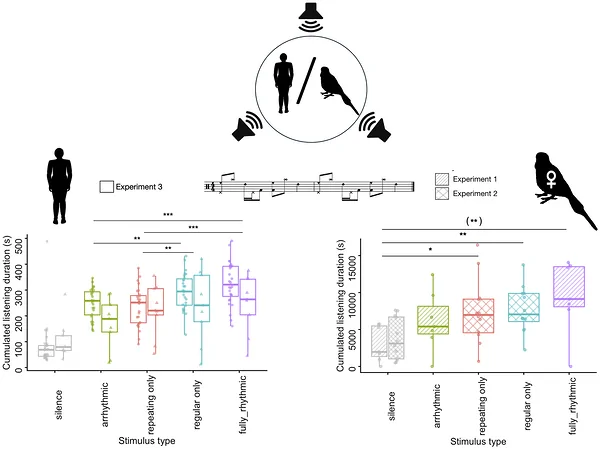

## 1 Introduction

Music is woven throughout our lives and across all human cultures, taking highly diverse forms—from lullabies for infants to community dances, hip hop, and free jazz improvisation. Yet, all music is based on biologically constrained cognitive capacities that enable us to produce and make sense of sound in specific ways. The set of these capacities has been termed *musicality* [1]. Culture both exploits and has shaped musicality throughout its evolution [2, 3], influencing musical‐acoustical features observed across cultures [4]. Probing the perception of such features can thus reveal cognitive capacities and their ecological relevance in human evolutionary history. This situates human music in the realm of animal communication and enables us to illuminate the biocultural evolution of music and musicality.

Two fundamental structural features of human rhythm are repetition and regularity. Repetition refers to the recurrence of patterns of sonic elements in a particular sequence [5]. Regularity refers to sonic events whose onset times are related by small integer ratios [6]. In Western music, for example, two eighth notes fit within one quarter note. Regularity and repetition are key components of other aspects of rhythm (e.g., groove perception [7]) based on entrainment, the ability to synchronize movement to music. Both regularity and repetition are cross‐cultural features of human music [4], although not universally present. In particular, whereas most human rhythms involve both repetition and regularity, a rhythm can be regular but not repeated (e.g., a drum solo with continuing variation of rhythmic patterns) or repeated but not regular (e.g., looped recordings of musique concrète). Why these features so often co‐occur in human rhythms remains unresolved. However, comparing musicality traits across species may illuminate this question.

Many animal taxa display rhythmic behaviors, including during locomotion [8], mating behavior [9], and in vocalizations [10, 11]. Some species show rhythmic behaviors resembling those in human music, such as entrainment (aligning movement period and phase to external sounds), isochrony (regularly timed sound events), or repetition of sound events or patterns. Notably, some parrot species spontaneously infer a beat from auditory sequences and entrain their movements to human music [12], resembling human groove behavior.

The capability of beat perception and synchronization has been hypothesized to be evolutionarily linked to vocal learning [13, 14]. Like humans, parrots are lifelong vocal learners [15]. Learning to produce and imitate vocalizations is a rare trait: Most animal vocalizations are innate [16]. Some parrot species also show nonvocal rhythmic behavior, such as head‐bobbing in courtship displays [9], and nonvocal movement imitation [17] resembling human dance. Humans and parrots also show similarities in their behavioral ecology. Vocal and rhythmic behavior as part of parrots’ social interaction can be highly rewarding to them [18]. This set of similarities makes parrots an important comparative model for studying the origins of auditory–motor entrainment. Comparing human and parrot responses to rhythm, both experimentally and in observing natural behavior, can thus help us understand auditory and social cognition of both and the evolutionary pressures that shaped this critical component of musicality.

One way to compare rhythm perception is through place preference studies [19] by measuring preference via listening duration. Unlike sound discrimination studies, preference studies can reveal which stimulus features are naturally rewarding or salient. In one such place preference paradigm, Hoeschele and Bowling [20] found that female budgerigars (*Melopsittacus undulatus*) preferred rhythmic over arrhythmic stimuli, similar to the human participants tested with the same paradigm, while male budgerigars showed the opposite pattern. Rhythmic stimuli consisted of regularly timed percussive sounds; arrhythmic stimuli were temporally jittered. This indicated that both female budgerigars and humans find sound events related by small‐integer ratios relevant, suggesting perceptual alignment across species.

However, the previous study could not disentangle whether females were attracted to rhythmic stimuli or repelled by arrhythmic stimuli (vice versa for male budgerigars). Resolving this requires an additional silence condition, avoiding a forced choice between sounds. We, therefore, added this silent option in the current studies. Because rhythmic stimuli in the previous study combined a repetitive pattern with a beat, while arrhythmic stimuli were randomized, it is unclear whether the underlying regular beat or the repetition of sound patterns drove the observed preferences. Humans appear to have a proclivity for beat inference in music based on perceiving hierarchical relationships [7], suggesting that attending to a regular beat is a hallmark of human rhythm perception. Whether this is the same for budgerigars—birds capable of audio‐motor entrainment [21]—and would translate into preferences for regularity over repetition is unclear. In the current study series, we, therefore, investigated this question explicitly.

We conducted three studies: experiment 1 extended Hoeschele and Bowling [20], using the same stimuli in a new three‐choice paradigm including a silent option. We predicted replication of Hoeschele and Bowling's findings: females listening longer to rhythmic and males to arrhythmic stimuli. We further hypothesized that this behavior would reflect attraction to the preferred stimulus, rather than avoidance of the dispreferred one (i.e., time listening to preferred > time listening to silence). In experiment 2, we tested the influence of repetition and regularity on rhythmic preference. We hypothesized that female budgerigars would prefer regular but nonrepeated stimuli over silence and possibly over nonregular but repeated stimuli. Experiment 3 examined human preferences for regular and repeated stimuli, or the combination of both, combining the conditions of experiments 1 and 2 in one study using the same three‐choice paradigm (including a silent option) to compare human and budgerigar preferences. We hypothesized that humans would prefer regularity over repetition. As musical experience often influences performances on acoustic tasks [22], we also assessed self‐reported musical experience. Although Hoeschele and Bowling [20] found no gender effects, we included this factor for comparability with budgerigars. Although tested conditions are distributed over two experiments for budgerigars, to our knowledge, this is the first study to explicitly compare preferences for rhythmic regularity and repetition between humans and budgerigars.

## 2 Experiment 1

### 2.1 Methods

#### 2.1.1 Subjects

We tested 13 adult budgerigars (seven females, six males), aged between 1 and 4 years. Birds were housed in the Animal Care Facility at the Department of Behavioral and Cognitive Biology, University of Vienna, in three mixed‐sex aviaries (two of size 2 × 1 × 2 m, one of size 2 × 1.5 × 2 m), each containing groups of 5–10 birds. A 12.5/11.5 h light/dark cycle was maintained, approximating their natural diurnal cycle [23]. Water, periodically supplemented with vitamin supplements and bird food pellets, was available ad libitum. None of the birds had prior experience with the testing apparatus, and the birds were naive to the current stimulus set. However, 10 of the birds (six females, four males) had participated in the Hoeschele and Bowling [20] study and, therefore, had experience with a different two‐alternative forced‐choice preference apparatus. All birds were accustomed to food‐rewarded operant conditioning tasks testing auditory cognition (during the period of the current studies: lexical stress [24] and octave equivalence [25]). All procedures involving animals in the studies described here were conducted in accordance with Austrian animal protection and housing laws and were approved by the ethical board of the behavioral research group in the faculty of Life Sciences at the University of Vienna (Approval Number 2015–005).

#### 2.1.2 Apparatus

Budgerigars were tested in a custom‐built hexagonal aviary that was acoustically isolated from the main aviaries in which they were normally housed. This testing aviary comprised three perches, each paired with an adjacent FE108 full‐range speaker (Fostex, Tokyo, Japan; frequency range: 200–16,000 Hz). Landing on a perch disrupted an infrared beam (IR Break Beam Sensor—3 mm LEDs, Adafruit Industries, New York, NY, USA), triggering stimulus playback from the speaker after 300 ms of beam disruption (according to a custom written Python script running on a MacBook Pro [Apple Inc., Cupertino, CA, USA] with an Arduino Uno REV 3 chip [BCMI, Turin, Italy] interface). Because pilot testing showed that birds would not interact with the apparatus when acoustically isolated from their flock, we played back 2 h recordings from their respective flock, at an intensity of approximately 50 dB SPL (measured at the bird's position) during testing sessions, using an AV 40 speaker placed above the testing apparatus (M‐Audio, Cumberland, RI, USA) controlled by an iPad (Apple Inc.). Water and food were available ad libitum on the apparatus’ floor throughout the experiment, not administered as rewards.

#### 2.1.3 Stimuli

**FIGURE 1:**
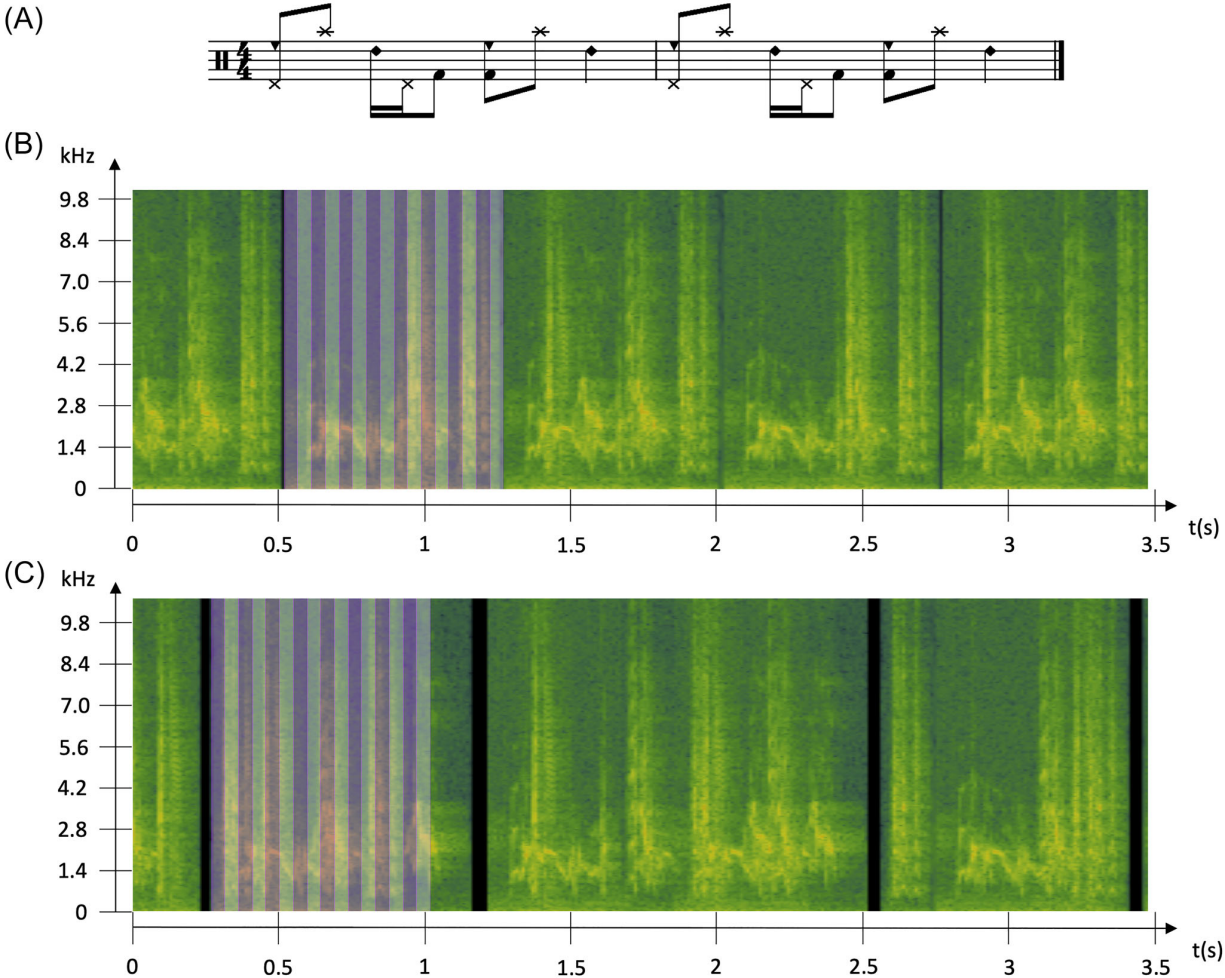
Sample stimuli used in experiment 1. (A) Illustration of rhythmic arrangement of the five budgerigar vocal units over two bars. Each note symbol/line represents a different vocal unit. (B, C) Spectrograms of the parts of sample stimuli for fully rhythmic (B) and arrhythmic (C) conditions. Purple overlays on spectrograms mark durations of 16th notes (one bar) underlying the creation of the respective stimulus. Created with Sonic Visualiser [64].

We created two sets of stimuli—fully rhythmic and arrhythmic—each set consisting of 600 stimuli, with a duration of 60 s per stimulus. Each stimulus was composed of five different budgerigar vocal units (∼100–200 ms in length), extracted from audio recordings of one adult (∼3 years) male budgerigar unfamiliar to the subjects here (one from Group C from [26], see recording details). Each stimulus was thereby composed of the same set of five vocal units, with the only difference between conditions being how they were arranged in time. Using a custom MATLAB script, we generated five rhythmic patterns. Each pattern consisted of five parallel tracks, one for each of the five different vocal units. To achieve this, we created a matrix representing two 4/4 bars, with possible sound onsets at each 16th note. Each pattern consisted of 20−26 events, with at least four events per track. One of the tracks had events on each quarter‐note beat to make sure there was a sound event on each beat in the final stimuli. This way, stimuli could be made rhythmic, such that inter‐onset intervals (IOIs) of subsequent vocal units were related by ratios of 1:1, 1:2, or 1:4 (see Figure 1). All of these rhythmic ratios were present in all stimuli; thus, a preference for specific rhythm ratios could not be tested with the current study design. The duration of each bar was approximately 1.5 s (160 bpm), resulting in about 40 repeating bars per 60‐s stimulus. Thus, fully rhythmic stimuli comprised both regularity and repetition. We then assigned all combinations of the budgerigar vocal units to the five tracks, resulting in 120 stimuli per rhythmic pattern (thus, 600 stimuli per condition). Stimuli were root mean square amplitude normalized and ramped (0.005 s fade in and out). Arrhythmic stimuli were created by introducing random jittering (within a ±200 ms window around the onset) to each of the individual rhythmic stimuli for a total of 600 arrhythmic stimuli. Stimuli were played at approximately 75 dB SPL at the bird's position.

To assess whether fully rhythmic and arrhythmic stimuli differed in acoustic dimensions other than the rhythmic manipulation, we compared stimulus sets for key acoustic features (see Supporting Information, pp. 2–4).

#### 2.1.4 Procedure

Birds received no training for the current study. After starting playback of the test bird's flock recording, the bird was introduced into the testing apparatus and remained there for 2 h. We tested each bird in three experimental sessions. The intersession interval was 1−9 days. For birds to be included in the sample, they had to listen to both sounds at least once across the three sessions, thus allowing them to make informed choices about listening. If a bird did not activate any of the perches in the first two sessions, we discarded these sessions and added a fourth or, if necessary, fifth session before a bird would be removed from the sample. All birds passed the criterion. Assignment of stimulus sets (fully rhythmic, arrhythmic, or silence) to perches was randomized across sessions and individuals. If a bird sat on the perch for at least 300 ms, a randomly selected stimulus from the appropriate set was played. If the bird sat longer than the duration of a stimulus, another randomly chosen stimulus from the same set began immediately. As soon as the bird left the perch, playback ended with a fade out of 50 ms. Across the session, stimuli were selected without replacement until all stimuli were played. During the study period, the same birds also participated in other operant conditioning studies (see above). Thus, birds were not naive to auditory experiments; however, they were naive to the stimuli used here. Additionally, since birds were not rewarded for the current and the previous (Hoeschele and Bowling's) [20] preference study on rhythm perception, we did not expect any biases. We also included “subject” as a random factor in our statistical models.

#### 2.1.5 Statistical Analysis

We first checked whether birds landed on each perch at least once across all sessions to confirm the possibility of informed choice. One female bird did not visit the silent perch; however, that bird generally spent very little time listening to the stimuli (< 10 s in total), probably spending most time at the floor or the walls of the testing apparatus, so we kept it in the sample, taking account for individual differences by including “subject” as a random intercept as well as random slopes across stimulus conditions in the statistical model. We modeled the effect of the birds’ preference for rhythmic versus arrhythmic stimuli versus silence using a linear mixed model with the cumulative duration spent listening to each condition and its interaction with the bird's sex as predictors. We first summed the durations each bird spent listening to each stimulus set across sessions to obtain our response. Because the response distribution was skewed, we then square‐root‐transformed these cumulated durations before fitting the model. All statistical analyses were done in R (version 4.4.0) [27]. We fitted the model using the function *lmer* (R package *lme4*; version 1.1‐30) [28]. Dummy coding for sex was done by a custom‐built function. We inspected a histogram and a qq‐plot of residuals and plotted residuals against fitted values but found no strong deviations from the assumptions of normality and homogeneity of residuals [29, 30]. We assessed collinearity by fitting a linear model without the random effect and interaction between fixed effects using the function *vif* (R package *car*; version 3.0‐2 [31]), which revealed no collinearity issues. Model stability was assessed by using a leave‐one‐subject‐out (LOSO, jackknife) sensitivity analysis for each random effect (see Supporting Information pp. 6–9 for details. R code generated with AI assistance), revealing the model to be reasonably stable. We obtained 0.95 confidence intervals using the function *bootMer* (1000 parametric bootstraps, package *lme4*, version 1.1–30 [28]; see Table S1). For each model, we report the marginal coefficient of determination ($R_{m}^{2}$), which reflects the variance explained by the fixed effects alone, and the conditional coefficient of determination ($R_{c}^{2}$), which reflects the variance explained by the fixed and random effects combined.

### 2.2 Results

The comparison of the full model with a null model devoid of fixed effects terms was not significant (*χ* ^2^ = 10.189, *df* = 5, *p* = 0.070, $R_{m}^{2}$ = 0.112, $R_{c}^{2}$ = 0.868). Thus, we could not conclude that the predictors of interest explained the data significantly better than the null model, although a trend could be argued (see Table S2 for estimated marginal means, R package *emmeans*, version 1.8.4‐1 [32]). However, given that we expected an interaction of preference with sex based on the previous study by Hoeschele and Bowling [20], we exploratorily compared the full model with a model reduced by the two predictors of interest (R function *drop1* with argument “test” set to “Chisq”) in a likelihood ratio test. This revealed no significant interaction between sound and sex (LRT = 4.798, *p* = 0.09).

**FIGURE 2:**
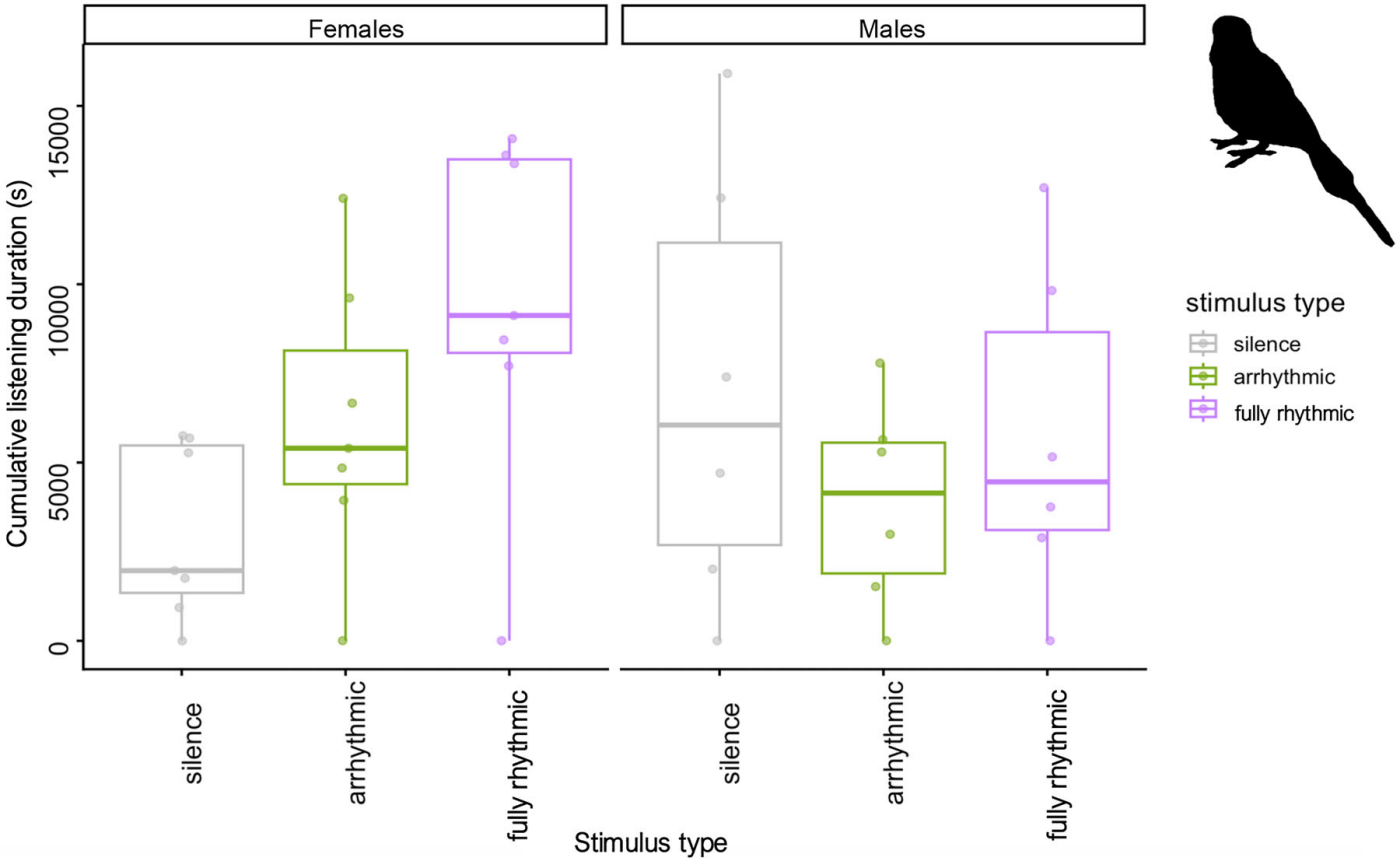
Data from experiment 1 (budgerigars) from the three‐place preference paradigm (silence, arrhythmic, and fully rhythmic stimuli). Listening durations (in seconds) to each stimulus type cumulated across sessions for each bird were used as the response variable to measure preference. Error bars represent IQR*1.5.

**FIGURE 3:**
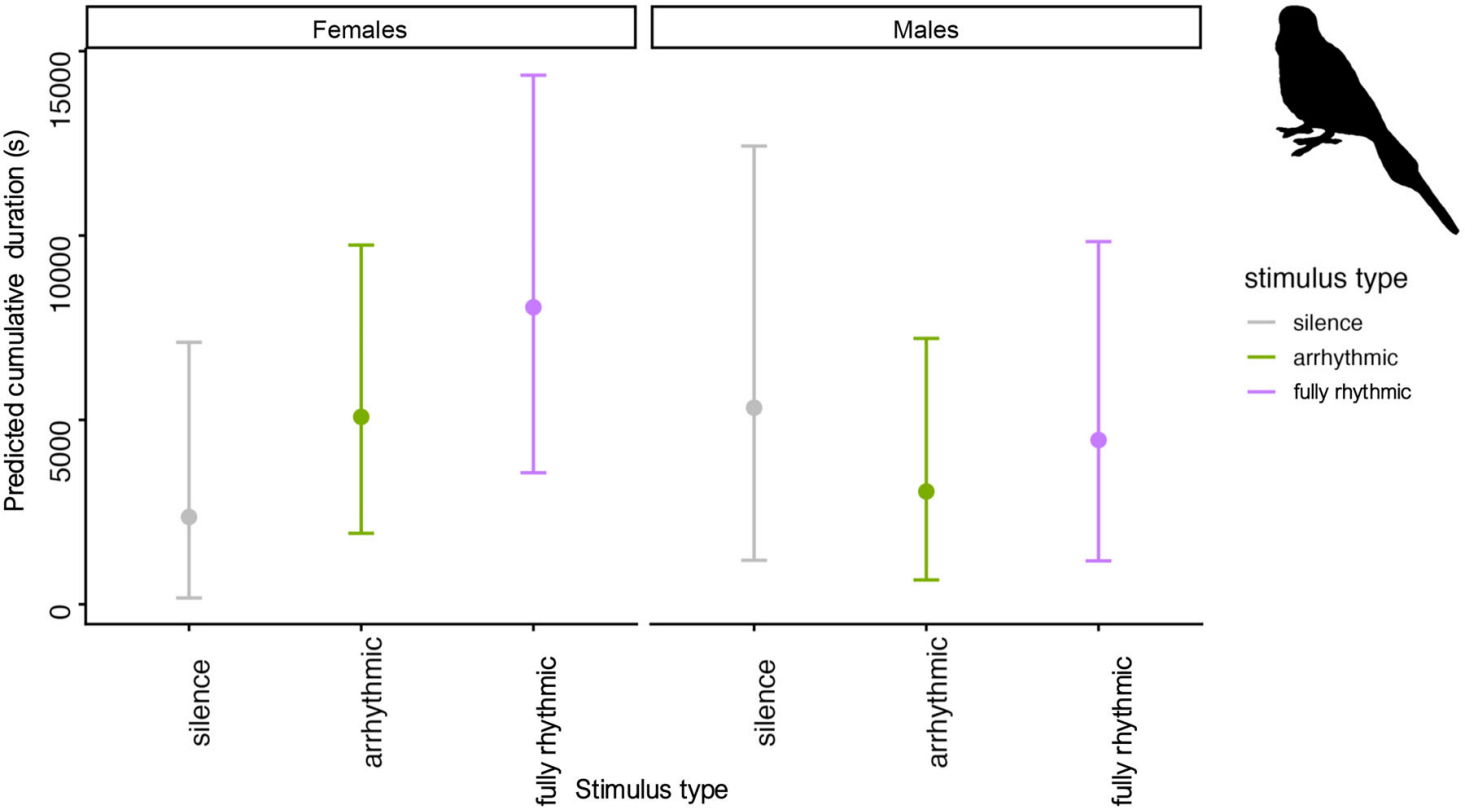
Experiment 1: Estimated marginal means of cumulative listening duration (responses transformed back to seconds) for budgerigars in the three‐place preference paradigm (silence, arrhythmic, and fully rhythmic stimuli). Points indicate model‐based estimates; error bars represent 95% confidence intervals.

Male budgerigars tended to prioritize silence over fully rhythmic and arrhythmic stimuli, while females tended to prefer fully rhythmic over arrhythmic and silence (see Figures 2 and 3), a finding that could be ecologically meaningful regarding the mating behavior of budgerigars, where males sing to females, including bouts of regular head bobbing (see Discussion). Although both sexes increased listening time for fully rhythmic as compared to arrhythmic stimuli, model estimates indicate that the gain for females was larger than for males. Exploratory analyses by sex supported this observation (see Supporting Information, p. 7).

Our acoustic analysis revealed a difference in amplitude entropy, likely resulting from temporal jittering in the creation of arrhythmic stimuli leading to more overlaps between vocal units, with more silent gaps and sharper amplitude peaks. We analyzed the stimuli to assess the influence of the acoustic metrics underlying the amplitude entropy difference.

Our results suggest that female budgerigars were both avoiding stimuli with high variation in amplitude in arrhythmic stimuli and preferring the higher local contrasts between IOIs of rhythmic stimuli (see Table S3).

#### 2.2.1 Bayesian Comparison of Hoeschele and Bowling's study and Experiment 1

The previous binary choice study [20] observed a significant effect of sex in rhythmic preferences, with females spending more time listening to fully rhythmic stimuli and males to arrhythmic stimuli. In the current three‐way choice study, we found the same general direction for female preferences. This suggests that there may be an underlying latent preference of females for choosing rhythm, for which both studies provide evidence under different designs, that is, under different data‐generating processes. To estimate evidence for this latent preference, given both studies, we analyzed listening behavior using a hierarchical Bayesian Dirichlet regression, modeling the proportion of total listening time each bird allocated to each stimulus category (R code generated with AI assistance). This approach allowed us to include both experiments despite different choice possibilities (binary choice between fully rhythmic and arrhythmic stimuli in Hoeschele and Bowling [20] vs. three‐way choice including silence in experiment 1), and thus different data‐generating processes.

Because the two experiments differed in the set of available response alternatives and, therefore, represent different data‐generating processes, we modeled each experiment separately with an otherwise identical hierarchical model structure and parameters. We calculated proportions of listening time per condition from the total listening time and included the fixed effects of sex, and random intercepts and slopes initially; however, due to low precision and high standard error, we decided to exclude the correlation between random intercepts and slopes for the final models to avoid overparameterization due to the small sample sizes. Since the Dirichlet distribution is bound between 0 and 1 and thus assumes nonzero values, we added a small constant (0.001) to instances when a bird did not spend any time listening to a stimulus condition.

We fitted all models using Hamiltonian Monte Carlo (R‐package *brms* [33]) (for model summaries, see Supporting Information, pp. 9–11). Since we observed some divergent transitions, we increased the target average acceptance probability to 0.99, resulting in no divergent transitions. R‐hats were at 1 for all model parameters. Trace plots indicated that chain mixing was good. Posterior predictive checks and scatter plots of observed versus predicted values suggested a reasonable fit for both models. Bayesian *R* ^2^ were high (Hoeschele and Bowling [20]: *R* ^2^ = 0.86, 2.5% CI = 0.462, 97.5% CI = 0.993 for both fully rhythmic and arrhythmic proportions; experiment 1: fully rhythmic: *R* ^2^ = 0.771, 2.5% CI = 0.437, 97.5% CI = 0.961; arrhythmic: *R* ^2^ = 0.747, 2.5% CI = 0.367, 97.5% CI = 0.960; silence *R* ^2^ = 0.900, 2.5% CI = 0.643, 97.5% CI = 0.988) and were affected by the small sample size and inclusion of the random intercept. For the data of Hoeschele and Bowling [20], the probability of direction (pd) indicated a chance of 82.1% that female, but not male, budgerigars reliably preferred fully rhythmic (proportion females: 73%, males: 55%) over arrhythmic stimuli. For experiment 1 (three‐choice paradigm), females’ proportion of choosing fully rhythmic (53%) was again higher than males’ (35%), with a 91.1% chance (pd) of a reliable pattern. The differences for choosing arrhythmic stimuli were smaller between sexes and less certain (females: 36%, males: 28%, pd = 74%).

**FIGURE 4:**
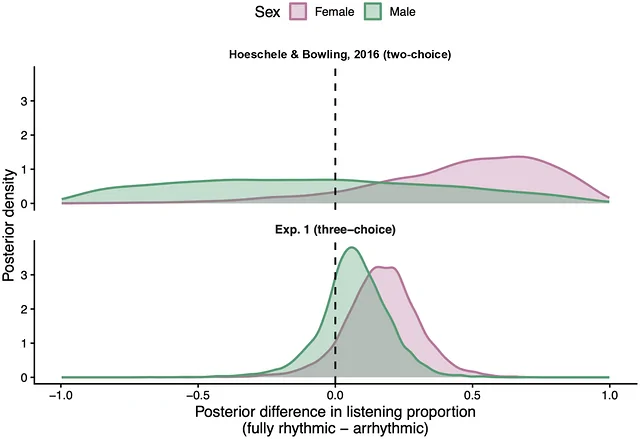
Posterior distributions of sex‐specific preference contrasts (fully rhythmic − arrhythmic) for Hoeschele and Bowling [20] (top) and the current experiment 1 (bottom). Dashed vertical lines indicate zero difference. Density plots illustrate posterior uncertainty rather than dichotomous significance.

We then computed posterior draws from each stimulus category and calculated mean differences and quantiles at 2.5% and 97.5%. We found that females allocated more listening time to fully rhythmic than arrhythmic stimuli in both Hoeschele and Bowling [20] (posterior mean difference = 0.462, 95% quantiles [−0.317, 0.921], P(Δ > 0) = 0.908) and in the current experiment 1 (mean difference = 0.169, 95% quantiles [−0.114, 0.433], P(Δ > 0) = 0.912). In experiment 1, females also showed a clear preference for fully rhythmic stimuli over silence (mean difference = 0.417, 95% quantiles [0.087, 0.618], P(Δ > 0) > 0.987). In contrast, posterior distributions of male budgerigars for a rhythmic preference were closer to zero in Hoeschele and Bowling [20] (mean difference = −0.095, 95% quantiles [−0.894, 0.809], P(Δ > 0) = 0.420); while males showed a directional preference for rhythm in the current experiment 1 (mean difference = 0.070, 95% quantiles [−0.204, 0.317], P(Δ > 0) = 0.755), indicating no consistent directional latent preference across both studies (see Figure 4).

To assess the evidence that female birds have a latent preference for rhythmic structure across both studies, we compared posterior contrasts for female birds. We found that the probability that data from both studies support a latent female preference for fully rhythmic over arrhythmic stimuli was 0.827. In contrast, the probability of males preferring fully rhythmic over arrhythmic stimuli, given the data of both studies, was 0.315. The probability of males preferring arrhythmic stimuli in both studies was even smaller (0.140).

#### 2.2.2 Summary

We observed that female budgerigars spent more time listening to fully rhythmic than arrhythmic stimuli. When splitting the data and exploring them separately for each sex, females were found to significantly prefer fully rhythmic but not arrhythmic stimuli over silence, while males mostly chose silence, without significant differences in preference. Taking the data of both Hoeschele and Bowling [20] and experiment 1 into account, the two experiments provide converging Bayesian evidence that female budgerigars preferentially attend to rhythmic structure, while male preferences appear inconsistent. Posterior estimates showed a consistent female bias toward fully rhythmic stimuli across both experiments. While uncertainty increased in the three‐choice paradigm, posterior mass remained strongly shifted toward fully rhythmic preference relative to both arrhythmic and silence. Our three‐place preference paradigm, therefore, suggests that the findings with Hoeschele and Bowling's [20] two‐place preference paradigm were likely driven by female preference.

Because we could not discriminate whether female birds’ preference for fully rhythmic stimuli was due to these stimuli being repeated or because they had an underlying regular beat, we decided to run a second study, testing a larger sample of female birds only with stimuli that pitted regularity against repetition. Given that some budgerigars in ten Cate et al. [34] appeared to distinguish regular from irregular sequences (when local features such as absolute time intervals between sound events were kept constant), as well as the results from [20], we hypothesized that regular stimuli would be preferred over silence. However, we did not have a specific hypothesis about whether regularity or repetition would be preferred.

## 3 Experiment 2

### 3.1 Methods

#### 3.1.1 Subjects

Twelve female birds from the same aviary groups as in experiment 1 participated in experiment 2. Five of these participated in experiment 1.

#### 3.1.2 Apparatus

The same apparatus as in experiment 1 was used.

#### 3.1.3 Stimuli

**FIGURE 5:**
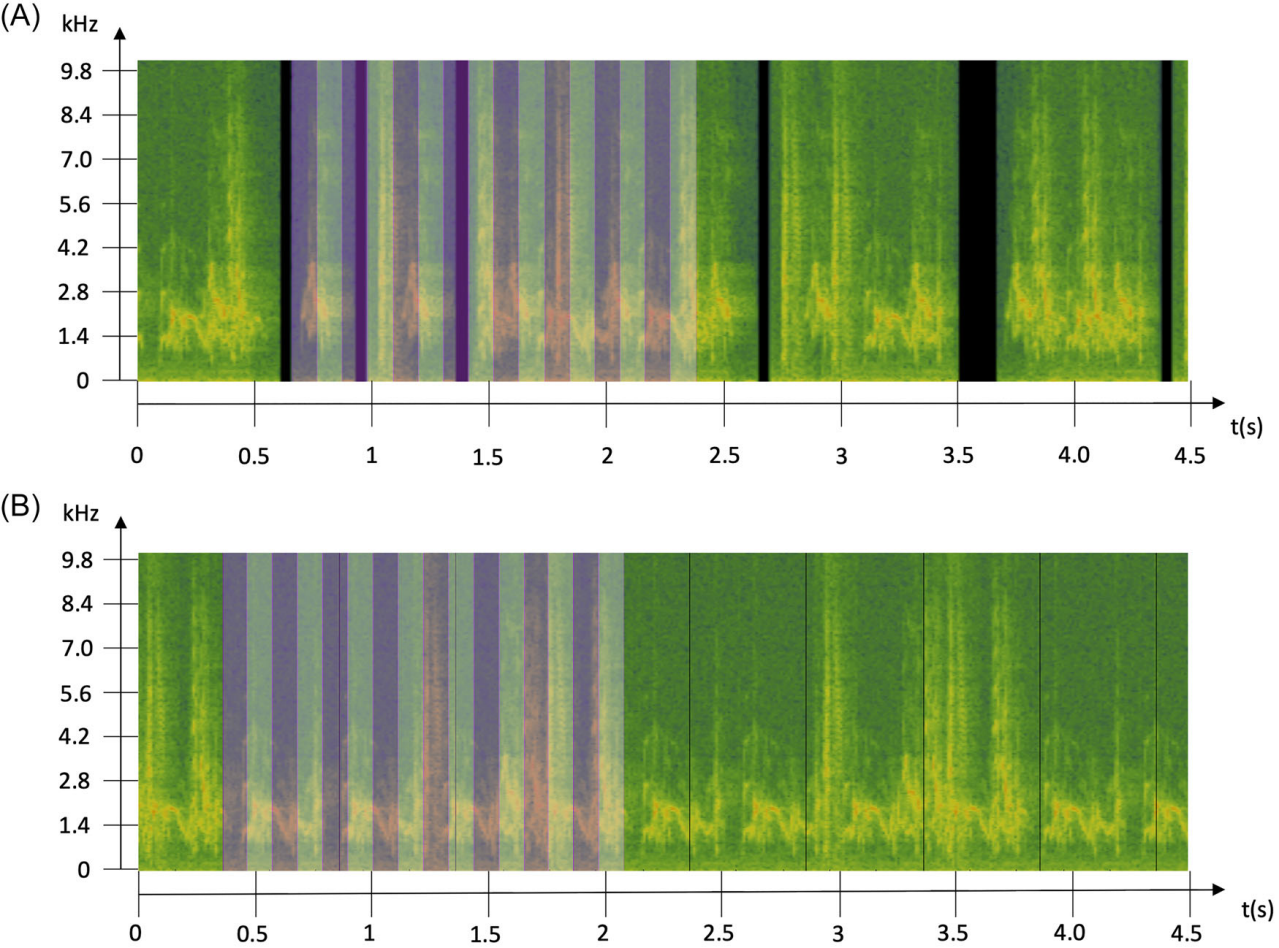
Spectrograms of parts of the sample stimuli used in experiment 2 (A) regular only, (B) repeating only. Purple overlays on spectrograms mark durations of 16th notes (one bar) underlying the creation of the respective stimulus. Created with Sonic Visualiser [64].

We created two new stimulus sets (see Figure 5) based on the same set of vocalization units from unfamiliar budgerigars used in experiment 1. “Regular only” stimuli were created in the same way as the fully rhythmic stimuli in experiment 1, but with a new pattern introduced every other bar rather than repeating the same two bar sequence (with 35−45 bars within each 1‐min stimulus, depending on the overall tempo), thus avoiding pattern repetition. For “repeating only” stimuli, the same two‐bar pattern was repeated, but with the onset times jittered such that the overall timing did not conform to a regular metrical grid. We used the same number of events (12 per bar) in both stimulus types.

In ten Cate et al. [34], budgerigars trained to discriminate regular from irregular patterns failed to generalize to novel stimuli that were changed in tempo. Because different tempos might be perceived differently and because effects confined to just one tempo may indicate preferences for absolute time intervals rather than generally for regularity versus repetition, each stimulus set here contained four 1‐min stimuli at 140, 150, 160, 170, and 180 bpm (= 20 stimuli per stimulus set). The duration of single bars was between 1.3 and 1.7 s, depending on the bpm. We again assessed acoustic differences between stimulus sets using the same analysis procedure and features as in experiment 1. We did not obtain any meaningful difference (probability outside ROPE > 0.95) for any acoustic feature (see Table S4).

#### 3.1.4 Procedure

The procedure was the same as in experiment 1. Birds were tested in three experimental sessions, each with a break of 1−7 days between sessions. The same birds also participated in operant conditioning studies between sessions. As in experiment 1, the assignment of stimulus sets to perches was randomized across sessions and individuals.

#### 3.1.5 Statistical Analysis

As in experiment 1, we used the cumulative perch duration per condition for each bird as the response variable, again square‐root‐transformed. All birds met the criterion of listening to each sound type (regular‐only, repeating‐only, silence) at least once, such that we did not have to exclude any bird's data. We used the same model parameters as in experiment 1, except without the factor *sex* and its interaction with sound, because we only tested female birds. Assumptions of normality and homogeneity of residuals were met per inspection of the distribution of residuals and residuals against fitted values. Stability analysis was done as in experiment 1, revealing a substantial influence of one bird (Melanzani, see Supporting Information pp. 12–14). We, therefore, ran a full model including this bird and a reduced model devoid of random slopes without Melanzani and report both results. We also analyzed post hoc whether those individuals who had participated in experiment 1 differed significantly from those who had not. This was not the case, suggesting that there was no effect of experience on preference choice (see Supporting Information, p. 1). The two stimulus sets did not differ across acoustic metrics (see Supporting Information p. 5).

### 3.2 Results

Full‐null model comparison revealed our initial model (including all birds) to explain the data significantly better than the null model (*χ* ^2^ = 10.670, *df* = 2, *p* = 0.005, $R_{m}^{2}$ = 0.216, $R_{c}^{2}$ = 0.846). The estimated cumulative amount of females’ listening time to either stimulus type (regular only: *m* = 7739 s, *SD* = 3127 s; repeating only: *m* = 7412 s, *SD* = 4396 s) was similar to that for rhythmic stimuli in experiment 1 (*m* = 9480 s, *SD* = 4961 s).

**FIGURE 6:**
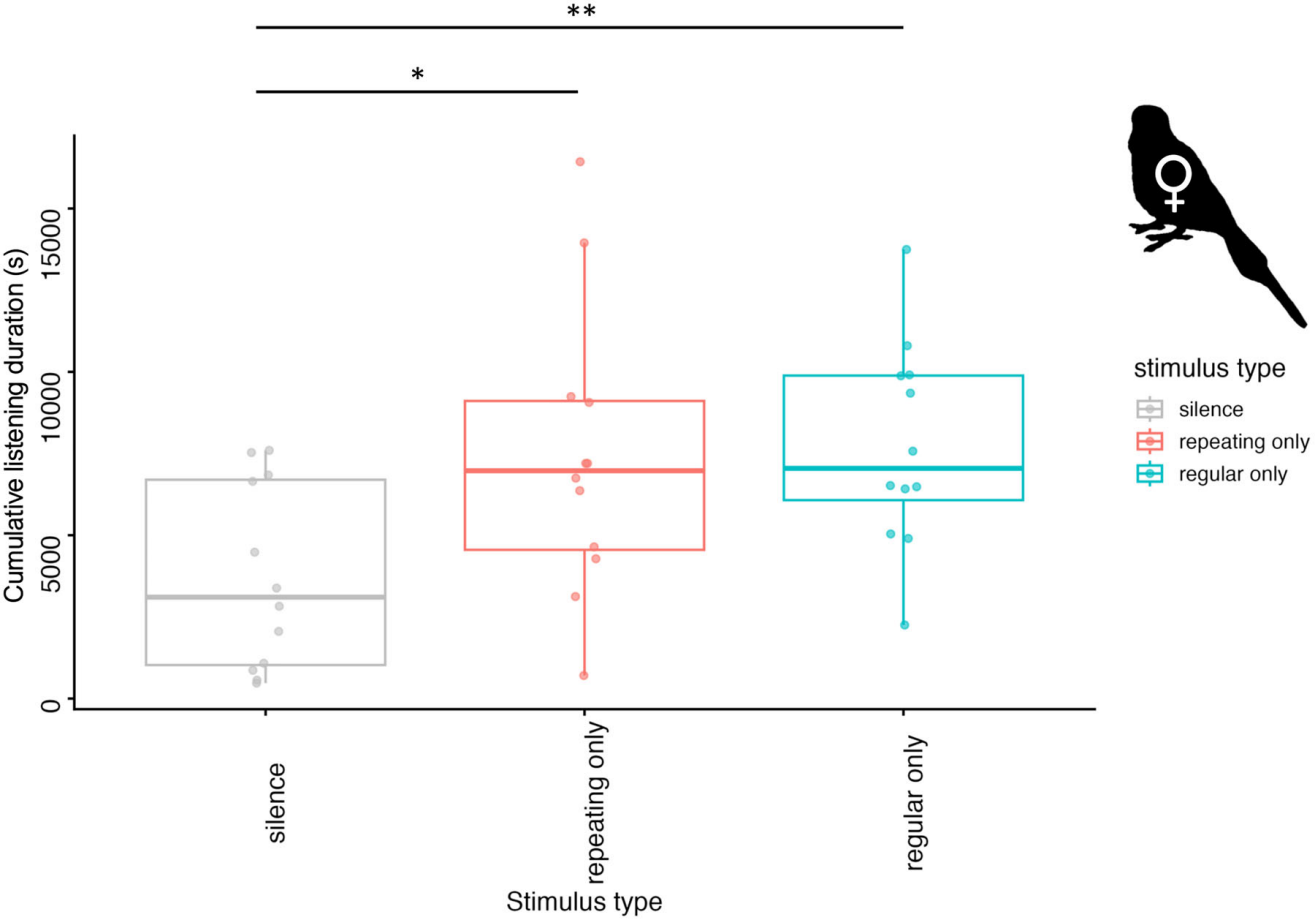
Data from experiment 2 (female budgerigars) from the three‐place preference paradigm (silence, repeating‐only, and regular‐only stimuli), including the bird Melanzani. Listening durations (in seconds) to each stimulus type cumulated across sessions for each bird were used as the response variable to measure preference. Error bars represent IQR*1.5. Significance was assessed by post‐hoc FDR‐corrected pairwise comparisons (R package *emmeans*, version 1.8.4‐1 [32]. Confidence level 0.95). Significance thresholds *p < 0.05, **p < 0.01.

**FIGURE 7:**
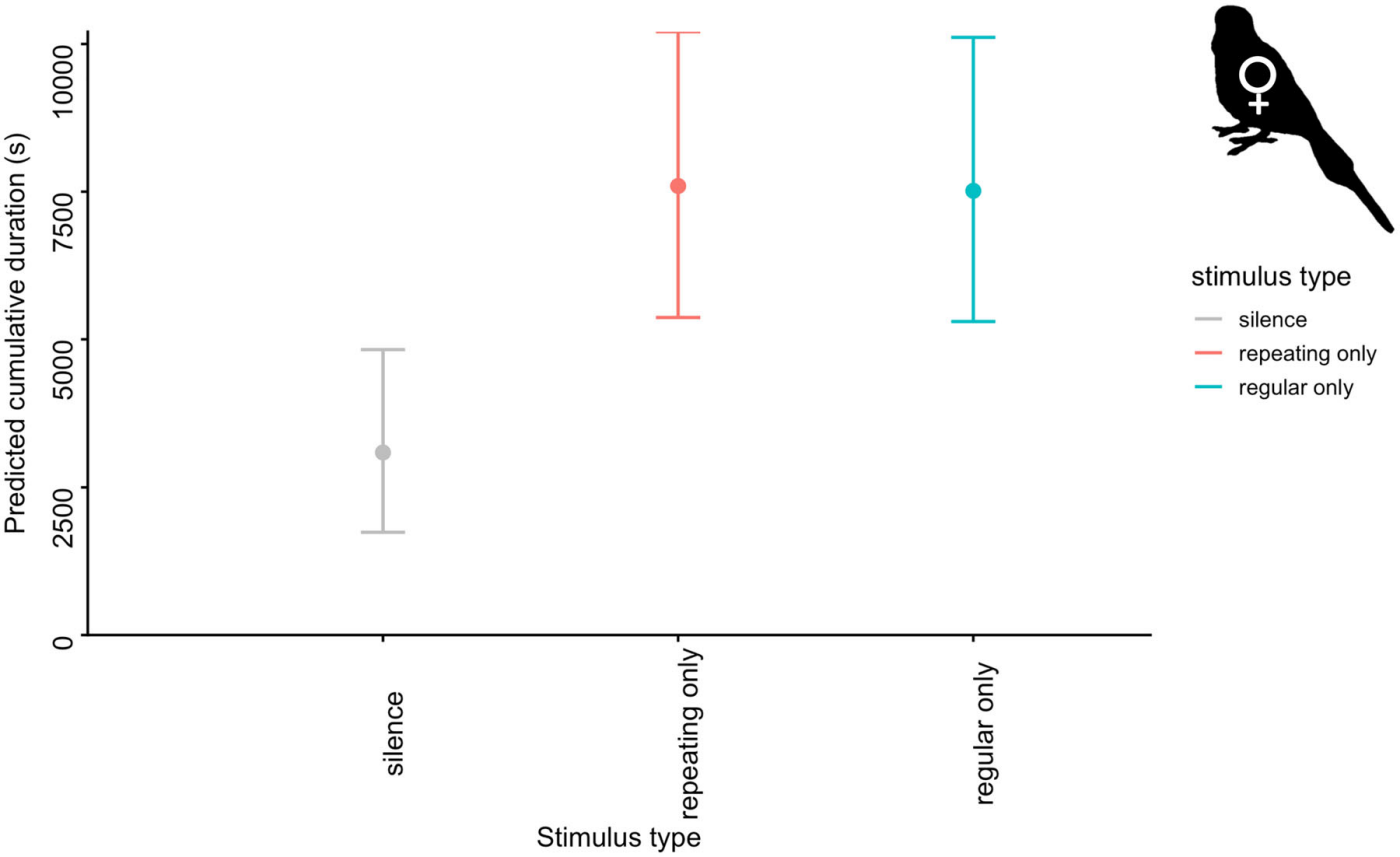
Experiment 2: Estimated marginal means of cumulative listening duration (responses transformed back to seconds) for female budgerigars (excluding the bird Melanzani) in the three‐place preference paradigm (silence, repeating‐only, and regular‐only stimuli). Points indicate model‐based estimates; error bars represent 95% confidence intervals. For the plot including the bird Melanzani, see Supporting Information.

Post‐hoc false discovery rate (FDR)‐corrected pairwise comparisons revealed that female birds spent significantly more time listening to repeating only (estimated marginal mean = 6741, estimated difference = 26.30, *SE* = 10.60, *df* = 15.9, *t*‐ratio = 2.480, *p*_FDR_ = 0.037) as well as regular only stimuli (estimated marginal mean = 7419, estimated difference = 30.33, *SE* = 6.64, *df* = 11.7, *t*‐ratio = 4.565, *p*_FDR_ = 0.002) compared to silence (estimated marginal mean = 3114). However, regular‐only and repeated‐only stimuli did not differ significantly in their listening time (estimated difference = 4.03, *SE* = 9.51, *df* = 11.9, *t*‐ratio = 0.424, *p*_FDR_ = 0.679) (see Figures 6 and 7). As the model excluding the bird Melanzani was a singular fit, we ran it without random slopes and with the random intercept only. The main effect of stimulus condition remained significant (*χ* ^2^ = 12.616, *df* = 2, *p* = 0.002, $R_{m}^{2}$ = 0.304, $R_{c}^{2}$ = 0.304), while the random intercept explained no variance without the bird Melanzani. Post‐hoc FDR‐corrected pairwise comparisons revealed very similar results to the initial model, but with strengthened effects and an inversion of the nonsignificant difference between the two sound conditions (regular‐only vs. silence: estimated difference = 31.093, *SE* = 9.68, *t*(20) = 3.212, *p* = 0.007; repeating‐only vs. silence: estimated difference = 31.565, *SE* = 9.68, *t*(20) = 3.261, *p* = 0.007; regular‐only vs. repeating‐only: estimated difference = −0.472, *SE* = 9.68, *t*(20) = −0.049, *p* = 0.9616), indicating Melanzani was masking the effects. Thus, our results were robust despite the small sample size and the outlier Melanzani.

#### 3.2.1 Summary

We found that female budgerigars significantly preferred listening to either repeating‐only or regular‐only stimuli as compared to silence but did not significantly prefer either stimulus type over the other. This suggests that both repetition and regularity are equally interesting for female budgerigars. Comparing the results to experiment 1, female birds spent a similar amount of time with repeating‐only and regular‐only stimuli as they did with the rhythmic stimuli comprising both components in experiment 1. We then decided to conduct the study in humans as well so that we could have a parallel cross‐species comparison. Both regularity and repetition are typical of music across cultures [4, 5]. Repeated rhythmic patterns allow for predictability, based on which surprising rhythmic deviations can occur [35]. Humans also have a proclivity to infer hierarchical meter from musical sequences, a process foundational for the perception of groove and for dance [7]. In turn, repetition without variation might lead to boredom due to lack of surprisal [36]. These observations suggest that humans gain more aesthetic appreciation from regularity over repetition, and potentially even more from the combination of both regularity and repetition.

## 4 Experiment 3

### 4.1 Methods

#### 4.1.1 Subjects

We tested 38 human participants aged between 18 and 43 years (9 males, 29 females, see Supporting Information p. 24). All participants gave informed consent to participate and were financially compensated with 5€ for every 30 min of participation duration (total duration ∼1 h). Participants were fully debriefed after the task and informed that they could object to the use of their data in the study at any time before publishing.

Experiment 3 was conducted in line with the Declaration of Helsinki and only employed methods that are covered by the standard ARI laboratory methodology that was approved by the Commission for Science Ethics of the Austrian Academy of Sciences on April 23, 2024.

#### 4.1.2 Setup

Participants were tested in an anechoic chamber of the Acoustics Research Institute (ARI) of the Austrian Academy of Sciences (Vienna). A designated circular test area within the anechoic room was demarcated with red tape on its periphery to prevent participants from interfering with setups for other studies in this room. Within this circumscribed space, three distinct locations were equidistantly placed with a distance of 120 cm from each other and visually marked by small black‐and‐white checkered mats with drawings of footprints on them to encourage participant interaction. A FE108 speaker (same type as was used in the bird studies) was located on a tripod in the center of the test area, about 70 cm from each mat (see Figure S20). Stepping on a mat for more than 300 ms triggered stimulus playback using an infrared beam interruption system, just like in the bird studies [1]. This was done using a custom‐written Python script and an Arduino Uno REV 3 chip; BCMI. Sounds were played from an HP Pro3500 Series computer (EXPBLAU‐ALT‐Intel Core i5‐3470 CPU). Similar to experiments 1 and 2, in each session of experiment 3, two mats triggered sounds from two different stimulus sets, while the third remained silent. We also monitored participants’ actions during experimental sessions using a preinstalled video surveillance system.^1^

#### 4.1.3 Stimuli

**FIGURE 8:**
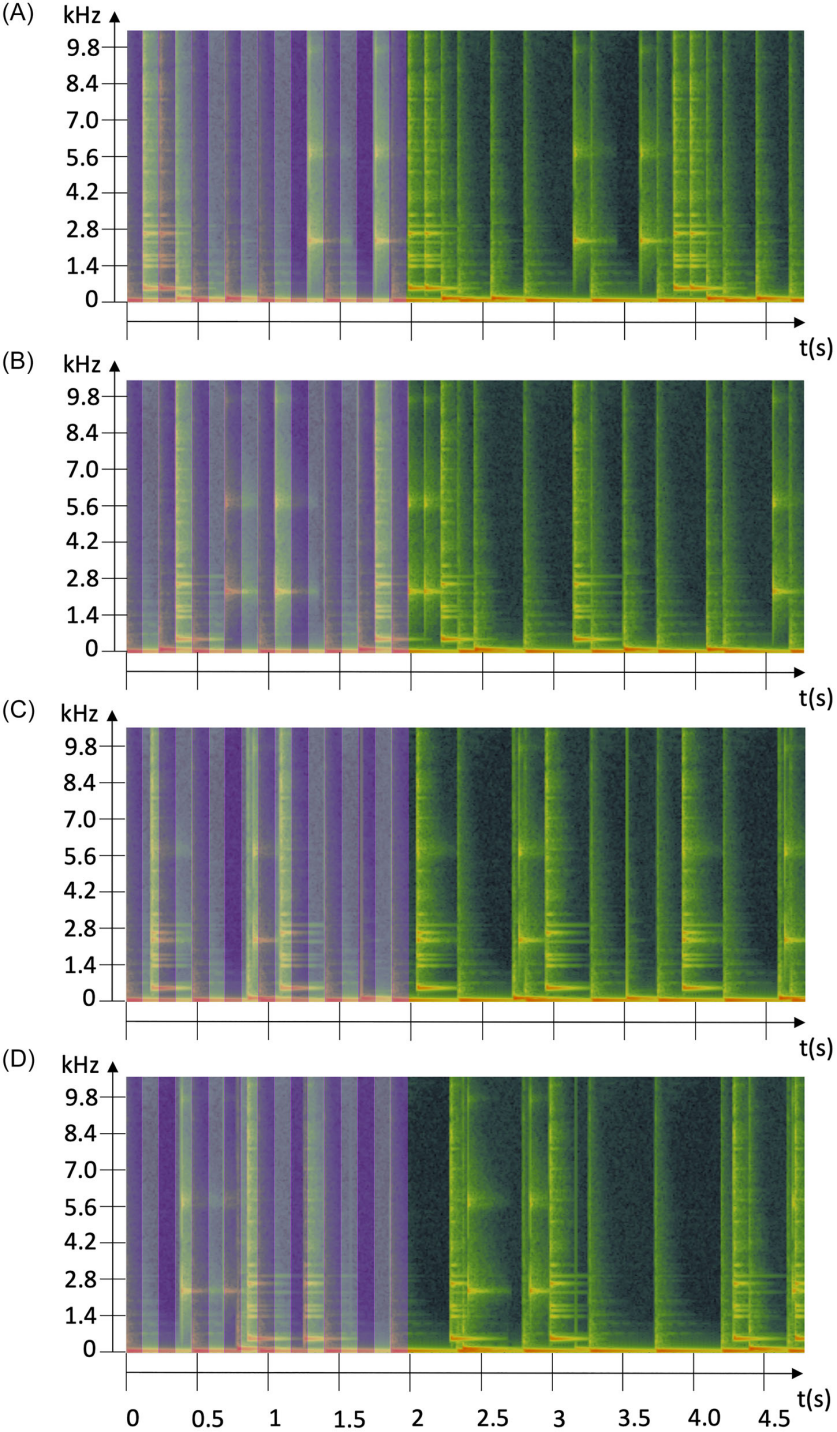
Spectrograms of parts of the sample stimuli used in experiment 3. (A) Fully rhythmic, (B) regular only, (C) repeating only, and (D) arrhythmic. Purple overlays on spectrograms mark durations of 16th notes (one bar) underlying the creation of the respective stimulus. Created with Sonic Visualiser [64].

We created four different sets of stimuli covering the same conditions used in both experiments 1 and 2—fully rhythmic, regular only, repeating only, and arrhythmic—but replacing budgerigar vocalizations with samples of five instruments from Logic Pro (version 9; Apple Inc.): shaker, clave, cowbell, djembe, and kick drum. Stimuli had a tempo of either 130, 140, 150, 160, or 170 bpm (4 stimuli per tempo, thus 20 stimuli per condition, resulting in 80 stimuli in total), 10 bpm slower than for budgerigars, respectively (see Figure 8). Stimuli were played at approximately 70 dB SPL at the participant's position. Note that because only two stimuli were played in each session (along with a silent choice), humans did both session types from experiments 1 and 2, as well as additional stimulus comparisons.

#### 4.1.4 Procedure

Participants were given written instructions in English explaining that their task was to explore a room in six 5‐min sessions. They were instructed to explore freely within an area around the speaker marked off with floor tape, with the added guidance that they could step on the three distinctly marked spots at their discretion. This guidance was designed to enhance comparability between the human and bird paradigms by encouraging participants to trigger the sounds (budgerigars naturally land on perches and rarely sit on the floor, the latter being comparable to stepping outside of the sound‐triggering locations here). Given that this study included four stimulus conditions (representing those used in both experiments 1 and 2), a Latin square design was used to balance the allocation of stimulus types across participants. This way, each participant could listen to each possible pairwise combination of stimulus types across their six sessions, while both the order of pairwise combinations as well as their assignment to the trigger locations on the floor differed between participants. Participants were allowed to take a break between sessions (max of 5 min) or continue immediately at their discretion. After the experiment, participants were asked to relisten to random sample stimuli from each stimulus set and rate them on 7‐point Likert scales (from “not at all” to “very”) for three questions: (1) How beautiful did you find the audio file?; (2) How interesting did you find the audio file?; and (3) How meaningful did you find the audio file?. Subsequently, participants filled out a questionnaire about their demographics (age, gender, etc.), and musical training (see Table S21 and Figure S19).

#### 4.1.5 Statistical Analysis

A linear mixed model was used to model participant‐level cumulative listening durations across sessions, as a function of stimulus condition in interaction with self‐reported years of musical training (max_instrument_years; when a participant learned several instruments, the longest training experience was taken) and stimulus condition in interaction with gender. We expected no further differences between genders with the same amount of musical training, thus no interaction of gender with musical training. Although the previous study by Hoeschele and Bowling [20] did not find an effect for gender, we included it for a more complete replication and comparison. We also included a random intercept for “subject” as well as random slopes. Our post‐experimental questionnaire showed that most participants had some amount of musical training (79% of 29 females, 67% of 9 males), next to a large skew in gender. We nevertheless included the interaction terms, keeping in mind a potential sampling bias toward females with musical training in our interpretation.

Model diagnostics revealed residuals to be homogeneous and approximately normally distributed, so no data transformation was necessary. We did not find any collinearity issues. The comparison of the initial full model with a null model containing only random intercept and slopes was highly significant (*χ* ^2^ = 142.514, *df* = 14, *p* < 0.001, $R_{m}^{2}$ = 0.484, $R_{c}^{2}$ = 0.606), providing omnibus protection against type I errors [37]. Due to stability issues, we simplified the initial model (see Supporting Information pp. 14–19). We report a final model, pruned of nonsignificant interactions with only main effects and the random intercept (cumdur ∼ sound + gender + max_instrument_years + (1 | subject)), which was statistically indistinguishable from the initial model.

### 4.2 Results

**FIGURE 9:**
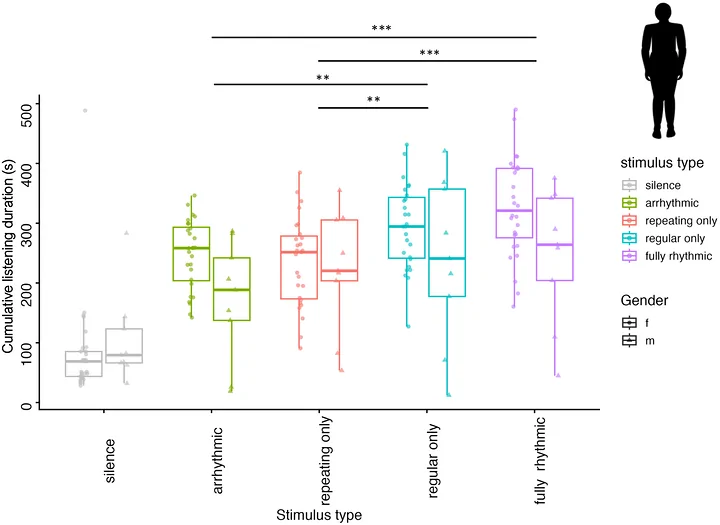
Data from experiment 3 (humans) from the three‐place preference paradigm (silence, arrhythmic, regular‐only, repeating‐only, and fully rhythmic stimuli; assignment to sessions and participants with a Latin square design). Listening durations (in seconds) to each stimulus type, cumulated across sessions for each participant (f, female, m, male), were used as the response variable to measure preference. Error bars represent IQR*1.5. Significance was assessed by post‐hoc FDR‐corrected pairwise comparisons (R package *emmeans*, version 1.8.4‐1 [32]. Confidence level 0.95). Significance thresholds *p < 0.05, **p < 0.01, ***p < 0.001. Significance for the main effect of gender and for contrasts of sounds against silence are not shown for spatial reasons (all significant, see Supporting Information).

**FIGURE 10:**
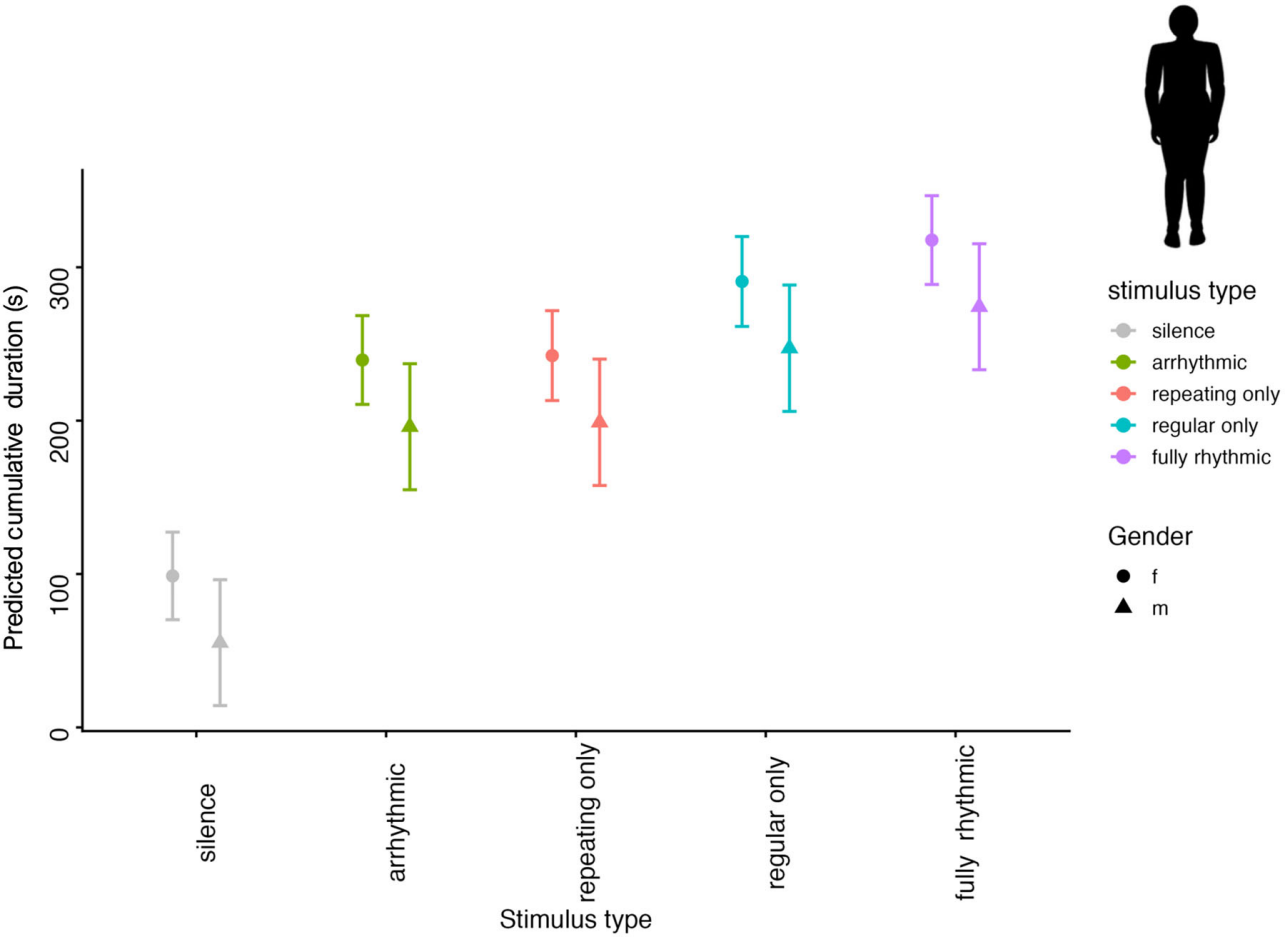
Experiment 3: Estimated marginal means of cumulative listening duration for human participants (f, female, m, male). Points indicate model‐based estimates; error bars represent 95% confidence intervals.

The full‐null model comparison was significant (*χ* ^2^ = 135.679, *df* = 6, *p* < 0.001, $R_{m}^{2}$ = 0.472, $R_{c}^{2}$ = 0.581). We found significant effects of stimulus condition (*F*(4,143.19) = 48.998, *p* < 0.001) and gender (*F*(1,34.04) = 4.676, *p* = 0.038) but not musical training (*F*(1,33.97) = 1.166, *p* = 0.288) (package *lme4*, version 2.0 6). We did FDR‐adjusted post‐hoc pairwise comparisons using estimated marginal means (package *emmeans*, version 1.8.4‐1 [32]). Participants preferred all sounds over silence, and fully rhythmic sounds were significantly preferred over both arrhythmic sounds (β = −78.01, *SE* = 17.2, *t*(142) = −4.523, *p* < 0.001) and repeating‐only sounds (β = −68.96, *SE* = 17.3, *t*(143) = −3.999, *p* < 0.001). Similarly, regular‐only sounds were preferred over arrhythmic (β = −54.70, *SE* = 17.2, *t*(143) = −3.172, *p* = 0.002) and repeating (β = 45.64, *SE* = 17.2, *t*(142) = 2.647, *p* = 0.011) conditions. There were no significant differences in listening duration between regular and fully rhythmic conditions (β = −23.31, *SE* = 17.2, *t*(143) = −1.352, *p* = 0.198) as well as between arrhythmic and repeating‐only (β = −9.05, *SE* = 17.2, *t*(143) = −0.525, *p* = 0.600) (see Figures 9 and 10). On average, female participants listened to the stimuli significantly longer than male participants (difference = 45.3, *SE* = 19.7, *t*(34) = 2.298, *p* = 0.028). Note that due to the instability of the previously found interaction, we can interpret this gender difference as being consistent across all sound types. A matched‐design analysis for comparability with experiment 2 also revealed a significant preference of regular‐only over repeating‐only stimuli (see Supporting Information, pp. 23–24). Post‐experimental questionnaire ratings were similar to the place preference results except for additional significance between fully rhythmic and regular‐only stimuli regarding perceived beauty and meaningfulness (see Supporting Information, pp. 21–22).

#### 4.2.1 Summary

We observed that humans preferred listening to fully rhythmic stimuli (combination of regular and repeating) and regular‐only stimuli over repeating‐only and arrhythmic stimuli. A similar pattern existed in the questionnaire responses regarding interest, aesthetic appreciation, and meaningfulness, with a significant preference for fully rhythmic stimuli regarding the latter two questions. This indicates large agreement between behavioral and self‐report measures of preference. Musical training did not exert a significant effect on listening preference, suggesting a low influence on human rhythm preference, which could be addressed in future studies.

## 5 Discussion

We investigated rhythmic perception preferences in budgerigars and humans, using a highly similar 3‐place preference paradigm. Although we did not find a significant effect for budgerigars’ rhythmic preferences based on our linear mixed‐effects model, incorporating data from both Hoeschele and Bowling [20] and the current experiment 1 provided converging evidence for a latent preference for rhythmic sounds in female budgerigars. In contrast, the pooled analysis revealed little evidence for male budgerigars for either rhythmic or arrhythmic latent preferences.

We found that both humans and female budgerigars preferred fully rhythmic over arrhythmic sounds. Both repetition and regularity of rhythmic patterns were attractive to listen to for both species (budgerigars in experiment 2; humans in experiment 3). When given the choice among all four stimulus types, humans preferred fully rhythmic and regular‐only stimuli the most. Since Hoeschele and Bowling [20] did not find a gender effect for humans, we interpret our observed gender effect cautiously, as our male sample was small (9 males, 29 females) and the initially observed interaction of gender and stimulus condition was unstable. We did not find a significant effect of musical training, suggesting it may be of lower priority for hypothesis‐generation in similar studies. Post‐experimental surveys largely confirmed the human results, with additional significance between fully rhythmic and regular‐only. This supports the validity of our extension of the place preference paradigm from birds to humans. Together, our results suggest that humans tend to prioritize a combination of regularity and repetition, while the presence of a regular beat seems to be the key factor.

For female budgerigars, both repetition and regularity (the small‐integer IOI‐ratio of subsequent elements) were equally attractive and preferred over silence, corresponding to both aspects characterizing rhythmic stimuli in Hoeschele and Bowling [20] and our experiment 1. In general, female birds spent more time listening to either stimulus type than males when having a silent choice, suggesting the stimuli were more interesting for females.

The ecology of budgerigars may explain these results. In contrast to Hoeschele and Bowling [20], we used vocal units from two male budgerigars instead of percussive sounds. Such units occur in budgerigars’ warble songs, which are mostly produced by males (rarely by females, see [38]), can be directed toward both males and females [39], and are used for social purposes in budgerigar colonies, such as communicating group identity [40]. Both sexes are, therefore, capable of producing and perceiving warbles, and accordingly, neurobiological sex differences in budgerigars regarding nonvocal motor and auditory pathways have not been established [41]. Thus, warble song units were expected to be interesting for both sexes.

However, warble song is most often produced by males in courtship toward females [42], with less interindividual variability than when directed at males [39]. Some researchers have, therefore, suggested that male warble song may be influenced by female preferences [39, 43]. Male budgerigars in turn engage in same‐sex courtship‐like behavior [44] and mate competition, suggesting that they assess other males’ warbles. Male budgerigars may, therefore, avoid other males that appear more dominant via their warble song. The observations in Hoeschele and Bowling [20] may thus reflect female interest in and (partial) male avoidance of the stimuli, a hypothesis testable using avoidance latency or physiological measures.

Female interest in warble song may in part account for their preference for rhythmic over arrhythmic stimuli. Natural warble song units can be locally repeated, sometimes with apparently regular onset times (see [40] and van der Aa et al. [11]). Budgerigars can discriminate regular and irregular strings of tones [45], suggesting they can perceive variability in regularity and repetition within warble song. Such variation may be meaningful for budgerigars, given warble song is influenced by female preferences [39, 43]. In the case of courtship, warble song units can also be accompanied by movements such as head bobbing, whereby certain unit‐movement associations can co‐occur more frequently than others [43]. Head bobbing is a repetitive behavior, and whether its frequency varies within a sequence could be investigated in future studies.

Thus, both head bobbing and local regular repetition of warble song units could increase the salience of regular and repeating patterns for females, motivating their listening preferences. Precise repetition or regularity may be perceived by females as more skillful and, therefore, more attractive. If so, male budgerigars with more rhythmic warbles should be more successful in mating. In turn, if males avoid each other in courtship, one would expect a decreased preference for rhythmic over arrhythmic stimuli (the opposite of what we observed in experiment 1, unless males learn from eavesdropping). This could be tested by analyzing natural warble structure in relation to mating success and hormonal changes and by manipulating courtship using an artificial bird with varying head bobbing and warbles (see, e.g., Mitoyen et al. [46]). Whether the preference for rhythmic sounds varies depending on environmental conditions favoring courtship could also be investigated.

One limitation is that rhythmic and arrhythmic stimuli in experiment 1 (but not in experiment 2, which controlled for sound overlap) differed in the amount of silent gaps and amplitude peaks (influencing amplitude entropy), a side effect of jittering onset times. Acoustic analysis suggested that female budgerigars preferred both a smoother amplitude peak sequences and sharp local IOI ratios, both direct correlates of our rhythmic stimuli. In experiment 2, female budgerigars significantly preferred both rhythmic stimulus types over silence, but not either of the rhythmic stimuli over the other. Thus, whether the stimuli were globally arranged in a metrical way or globally repeating, it does not seem to matter to the budgerigars as long as high local timing contrasts are embedded in a smooth amplitude stream. Notably, all budgerigar stimuli contained sound overlaps, similar to natural budgerigar flocks where many birds vocalize concurrently and produce overlaps interspersed with silent gaps. Thus, natural sound patterns may be closer to our arrhythmic than rhythmic stimuli. Given the observed preference patterns, birds may focus their attention on vocalizations of specific individuals within the flock to fulfill their preferences for a smooth amplitude stream with local timing contrasts, a hypothesis inviting future testing.

A further caveat is that female birds were not exposed to all four stimulus sets in one experiment, unlike humans. We thus lack direct evidence of whether female birds would choose fully rhythmic over regularity‐only or repeating‐only, though absolute listening durations across experiments 1 and 3 suggest they would. Analyzing human sessions with just repeating‐only and regular‐only stimuli shows that, unlike female budgerigars, humans tended to have a stronger motivation to listen to stimuli containing a regular beat (see Supporting Information pp. 23–24). These differences between humans and budgerigars may correspond to their behavioral ecology: while warble songs and head bobbing are solo displays, human music making is mostly a group activity across cultures [47]. In humans, concurrent sound production in groups (see [48]) requires predicting sound events with sufficient precision. Creating a beat using regularity would enable this. Combined with some level of surprise, music exploiting the sweet spot of predictability [49] is perceived as rewarding and fuels musical engagement [36, 50]. Perceiving hierarchical meter requires a shared predictable frame over which surprising sound events can be placed [51]. That we did not observe a significant effect of musical training suggests that everyday music exposure may suffice to induce such preferences, a hypothesis to be addressed in future research.

Relatedly, experience with chorusing or ensemble music may enhance preferences for regularity. To test this in humans, we can explore cross‐cultural variability since neither regular meter nor phrase or motivic repetition exist in all forms of music across cultures [4]. Thus, even if perceiving and producing regularity and repetition is part of human cognition, motivational and aesthetic aspects may depend on cultural exposure. The development of such preferences in children of different cultures may be especially illuminating [52]. Our paradigm can be easily adapted to cross‐cultural testing of musical preferences and to children from the crawling stage, thereby illuminating the interplay of biological traits, behavioral ecology, and development.

This aspect of human behavioral ecology—group chorusing allowing for both synchronous sound production and deviations within a predictable frame—has not been observed in budgerigars. Instead, our observed sexual dimorphism suggests that both regular and repeating stimulus types indicate skillful and, therefore, attractive mates. Thus, despite shared vocal learning and entrainment skills and functionally similar neurophysiology [13, 14, 15], perceptual preferences may depend on species‐specific behavior exploiting biological traits to different ends. As we did not directly compare budgerigars’ preferences for fully rhythmic versus regular‐only and repetition‐only stimuli, we cannot draw the same conclusions as for humans. However, the human results suggest that both entrainment and natural chorusing behavior would affect preferences for regularity, regarding variability both between and within different species. We, therefore, hypothesize that species engaging in chorusing should prefer regularity over repetition, at least if they are entrainers and/or vocal production learners. Thus, training budgerigars or other species to chorus and investigating the resulting preference changes for regular versus repeating stimuli may be illuminating. In turn, variability in local regularity and repetition in courtship displays may also influence rhythmic preferences. Thus, future studies could expose budgerigars to regular or repetitive warble songs in socially relevant contexts (see, e.g., [46]). Our paradigm can easily be adapted to other species to implement such testing. Investigating different species varying in vocal learning [53], entrainment [14], and chorusing behavior (e.g., plain‐tailed wrens [48, 54]) could help to estimate the influences of traits and ecology on perceptual preferences.

An alternative explanation for human preferences for regularity concerns syntax. Regularity as an important aspect of human musicality [7, 55, 56] may be related to the human proclivity for inferring hierarchical structures [57]. Cross‐culturally, most kinds of music show hierarchical regularity [4]. The ability to align note onsets on beat subdivisions in a musical sequence may require supra‐regular syntactic abilities (see [58, 59]). Although budgerigars can entrain to a musical beat, they seem to process sequences mostly based on surface features [60], largely restricted to two‐element relationships. Warble songs can be described as a fifth‐order Markov sequence [42, 61, 62], corresponding to finite‐state grammars. Thus, human preferences for regularity may reflect their ability to parse hierarchical meter, a syntactical depth likely irrelevant for budgerigars (see [63]). Since we did not observe a significant effect of musical training, everyday musical (and likely linguistic) exposure to exploit such biological traits seems to suffice to shape perceptual preferences. This interaction between evolutionary constraints and behavioral motivation may help explain differences in rhythmic capacities across vocal learning species.

## 6 Conclusion

The current series of studies is the first to show parallels in preferences for regularity and repetition in humans and budgerigars. That both female budgerigars and female humans are attracted to both regularity and repetition as two important aspects of rhythm perception shows parallels between distantly related species that have not previously been studied. These results suggest that species that are able to entrain to a beat may also share deeper parallels in the aesthetic motivations driving these abilities. The influence of behavioral ecology on the preference for these aspects as well as on the possible evolutionary refinement of traits for rhythm perception are exciting directions for future research. Our paradigm can be adapted to cross‐species, cross‐cultural, and developmental research to this end.

## Author Contributions

F.H.: Writing – original draft, Writing – review and editing, statistical analysis, building of human apparatus, co‐supervision of M.A. D.L.B.: Conceptualization, experimental design, stimulus creation, Writing – review and editing, co‐supervision of S.A. and J.K. S.A.: Building and programming of budgerigar apparatus, conducting experiment 1. J.K.: Stimulus creation, conducting experiment 2. M.A.: Modification of human apparatus, conducting experiment 3. M.H.: Conceptualization, experimental design, main supervision, Writing – review and editing, resources.

## Conflicts of Interest

The authors declare no conflicts of interest.

## Supporting information




**Supplementary Information**: nyas70389‐sup‐0001‐SuppMat.docx

## Data Availability

The data that support the findings of this study are available at https://osf.io/mj4kp/overview.

## Footnotes

1 We opted to mark designated areas and use video surveillance because, during pilot experiments, participants began interacting with other devices in the test room after being instructed to explore the space freely without further guidance.
