# Supplementary material for: Female Budgerigars Attend Equally to Rhythmic Beat or Repetition, While Humans Prioritize Their Combination
Source: Ann N Y Acad Sci. 2026 Sep 22;1563(1):e70389. doi: 10.1111/nyas.70389 (PMC13596745; doi:10.1111/nyas.70389)
Supplement: Supplementary file 1 [file NYAS-1563-0-s001.docx]

**Information for Female budgerigars attend to musical beat and repetition, humans prioritise their combination**

Felix Haiduk^1,2,5^*, Daniel L. Bowling^3,4,5^, Shahrzad Afroozeh^5^, Jan Kopač^5^, Mahin Azarian^1,5^, Marisa Hoeschele^1,5^

1 Acoustics Research Institute, Austrian Academy of Sciences, Vienna, Austria

2 Department of General Psychology, University of Padua, Padua, Italy

3 Department of Psychiatry and Behavioral Sciences, Stanford University School of Medicine, Stanford, CA, USA

4 Center for Computer Research in Music and Acoustics, Stanford University, Stanford, CA, USA.

5 Department of Behavioral and Cognitive Biology, University of Vienna, Vienna, Austria

Corresponding author: felix.haiduk@oeaw.ac.at

Appendix on AI use

We used a vibe coding approach using GPT-5 (OpenAI) and Google Gemini (accessed February 2026) to generate and refine R code for model stability analysis and plots, as well as for acoustic feature comparisons (control analysis), and the Bayesian analysis involving experiment 1 and 2 (including troubleshooting), during the revision of this manuscript. Prior to submission of the revised manuscript, we reviewed the content generated and take full responsibility for the content of the submitted manuscript.

Effect of experience of birds participating in both Exp. 1 and Exp. 2

Because 5 female birds that participated in Exp. 1 also participated in Exp. 2, we were concerned that there might be carryover effects. However, this was not the case and so we did not include our analyses in the main paper. Here we summarize these analyses in more detail.

To assess whether there was an effect of these birds’ experience on preference choice behavior we modeled cumulated listening duration as a function of the interaction between stimulus condition and a bird’s participation in Exp. 1. We included random intercepts and slopes for each bird. The full-null model comparison was significant (χ2 = 13.272, df = 5, p = 0.02, R2m = 0.246, R2c = 0.826). Both the interaction (LRT=0.508, p=0.776) and the main effect of participation in Exp. 1 (LRT=2.094, p=0.148) were not significant. Thus, we cannot conclude that experience in Exp. 1 had an effect on preference choice behavior.

Figure S1: Spectrograms of budgerigar vocal units that were used to create stimuli for experiments 1 and 2. Created with Sonic Visualiser ^1^. Note: names for vocal units were chosen for easier discrimination and are not based on standardized terminology in the literature.

Control of acoustic features between stimulus sets

Although stimuli for both conditions in Exp. 1 as well as Exp. 2, respectively, were created from the same set of budgerigar vocal units, differing only in their temporal arrangement, we conducted acoustical comparisons between both stimulus sets within each experiment to make sure effects depended on our manipulation and not unintended acoustic differences between stimulus sets. We used Bayesian Gaussian regression models for key acoustic features in frequency, spectrum and amplitude. For each feature, we estimated 95% credible intervals and posterior probabilities of differences between stimulus sets. We then assessed whether observed acoustic differences were perceptually meaningful for budgerigars. We defined regions of practical equivalence (ROPEs) for each acoustic measure, informed by psychoacoustic studies on budgerigar/avian hearing ^2–7^. These bounds reflect differences unlikely to be perceptually distinguishable by budgerigars under natural conditions, and a high posterior probability that differences were below perceptually meaningful thresholds. For most acoustic features, the majority of posterior mass lay within ROPE bounds, indicating practical acoustic equivalence between stimulus sets and suggesting that unintended acoustic differences are unlikely to account for the observed behavioral preferences (see Table S2). However, amplitude entropy differed between rhythmic and arrhythmic stimuli, with higher entropy in rhythmic sequences. We suspected this to be an artifact of jittering, since this might have led to overlaps of sound units with higher amplitude peaks and more silent gaps as compared to stimuli arranged on a metrical grid, resulting in a broader amplitude distribution. To assess this possibility, we calculated the proportion of samples below a threshold of -60 dB as a proxy for silent gaps for both stimulus sets. Using a Wilcoxon test revealed that the arrhythmic stimulus set had a significantly higher proportion of silent gaps (M=13.5%, SD=3.2%) than the rhythmic stimulus set (M=10.6%, SD=4.8%) (W=247104, p<0.001, Cohen’s d=0.719). We also calculated metrics regarding amplitude peaks (“find peaks” function from scipy.signal, prominence=0.05, distance=0.03s). We observed a significantly higher amount of amplitude peaks in the fully rhythmic stimulus set (median=920) than the arrhythmic stimulus set (median=857, Mann-Whitney-U test: U = 246918.5, Z = 11.212, p<0.001, r = -0.374) as well as significantly higher values in the arrhythmic than fully rhythmic stimulus set in average peak prominence (fully rhythmic median=0.137, arrhythmic median=0.141, U = 130518.0, Z = -8.204, p<0.001, r = 0.274), peak-to-average power ratio (fully rhythmic median=21.042, arrhythmic median=22.834, U = 2688.0, Z = -29.527, p<0.001, r = 0.985) and coefficient of variation of inter-peak intervals (fully rhythmic median=0.706, arrhythmic median=0.865, U = 76857.0, Z = -17.155, p<0.001, r = 0.572). This suggests that temporal jittering in the creation of arrhythmic stimuli resulted in more overlaps between vocal units, with more silent gaps and sharper amplitude peaks, explaining the difference in amplitude entropy. We then analyzed the stimuli to assess the influence of the acoustic metrics underlying the amplitude entropy difference. We calculated the normalized pairwise variability index (nPVI, ^8^), measuring alternation of adjacent time onset intervals, based on IOIs derived from amplitude peaks. We found this measure to be significantly higher in the set of fully rhythmic (mean=62.579) than arrhythmic stimuli (mean=60.197, t=-7.439, df= 745.34, p<0.001), the former containing sharp ratios (1:1,1:2,1:4) which were blurred through the temporal jitter when creating the arrhythmic stimuli. We also calculated IOI entropy and found the reverse pattern (fully rhythmic mean=0.678, arrhythmic mean= 0.820, t=26.820, df= 852.55, p<0.001). To obtain which of the calculated parameters best explained listening duration for female budgerigars, we applied model selection, starting with a linear mixed model including average prominence, peak-to-average power ratio, coefficient of variation of inter-peak intervals, nPVI and IOI entropy, as well as a random intercept for subject. For the model selection of acoustic metrics we found that the best model retained peak-to-average power ratio (sum of weights = 1.00), nPVI (0.99) and coefficient of variation of inter-peak intervals (0.82).

Here we summarise the results of these comparisons.

Table S1: Features and ROPEs used

| Feature | ROPE |
| --- | --- |
| mean f0 | ±5 Hz |
| f0 SD | ±5 Hz |
| mean spectral centroid | ± 15 Hz |
| mean spectral bandwidth | ± 15 Hz |
| spectral rolloff (85%) | ± 15 Hz |
| N spectral peaks | ±1 peak |
| spectral flatness | ±0.05 |
| amplitude entropy | ±0.05 |
| Budgerigar-specific MFCC Volatility | ±0.5 (z-units*) |
| mean RMS | ±0.01 |
| RMS SD | ±0.01 |
| mean zero-crossing rate | ±0.01 |

*Budgerigar-specific MFCC was calculated based on critical ratios from ^3^. We used the normalized variance of resulting MFCC coefficients to prevent loudness (coefficient 1) from dominating. We then z-standardized to have ROPE bounds defined in standard-deviation units.

Table S2: Results for arrhythmic vs fully rhythmic stimuli (Exp. 1) (R code generated with AI assistance). Interpretation as: probability in rope >0.95: "Practically equivalent", probability outside rope >0.95: “Meaningful difference”, else: inconclusive

| Feature | mean difference between stimulus sets | lower CI (0.025) | upper CI (0.975) | Probability in ROPE | Interpretation |
| --- | --- | --- | --- | --- | --- |
| amplitude entropy | 0.317 | 0.302 | 0.333 | 0.000 | Meaningful difference |
| N spectral peaks | 1.140 | 0.587 | 1.699 | 0.304 | Inconclusive |
| f0 SD | -4.221 | -5.984 | -2.507 | 0.810 | Inconclusive |
| f0 mean | 1.877 | -0.006 | 3.799 | 0.999 | Practically equivalent |
| Budgerigar-specific MFCC Volatility (z-units) | -0.100 | -0.218 | 0.020 | 1.000 | Practically equivalent |
| mean RMS | 0.001 | 0.001 | 0.001 | 1.000 | Practically equivalent |
| RMS SD | -0.001 | -0.001 | -0.001 | 1.000 | Practically equivalent |
| mean spectral bandwidth | -0.206 | -2.133 | 1.797 | 1.000 | Practically equivalent |
| mean spectral centroid | 0.657 | -1.281 | 2.566 | 1.000 | Practically equivalent |
| spectral rolloff (85%) | -0.104 | -2.071 | 1.807 | 1.000 | Practically equivalent |
| spectral flatness | -0.018 | -0.021 | -0.015 | 1.000 | Practically equivalent |
| mean ZCR | 0.004 | 0.003 | 0.004 | 1.000 | Practically equivalent |

Table S3: Model summary of best fitting model in the model selection of the influence of acoustic parameters on listening duration in female budgerigars (best model: duration ~ CV_Intervals + nPVI + PAPR_dB + (1 | subject))

| Term | Estimate | SE | df | t | p |
| --- | --- | --- | --- | --- | --- |
| (Intercept) | 44.4748 | 8.0756 | 5.8448 | 5.507 | <0.01 |
| CV_Intervals | 0.7013 | 0.2938 | 5102.1071 | 2.387 | <0.05 |
| nPVI | 0.8670 | 0.2384 | 5102.0296 | 3.637 | <0.001 |
| PAPR_dB | -3.3315 | 0.2937 | 5102.0980 | -11.342 | <0.001 |

Table S4: Results for repeating-only vs regular-only stimuli (Exp. 2) (R code generated with AI assistance). Interpretation as: probability in rope >0.95: "Practically equivalent", probability outside rope >0.95: “Meaningful difference”, else: inconclusive

| Feature | mean difference between stimulus sets | lower CI (0.025) | upper CI (0.975) | Probability in ROPE | Interpretation |
| --- | --- | --- | --- | --- | --- |
| amplitude entropy | -0.033 | -0.153 | 0.090 | 0.522 | Inconclusive |
| N spectral peaks | -0.164 | -1.909 | 1.544 | 0.735 | Inconclusive |
| spectral flatness | 0.031 | 0.000 | 0.062 | 0.894 | Inconclusive |
| mean ZCR | -0.004 | -0.008 | 0.000 | 0.999 | Practically equivalent |
| Budgerigar-specific MFCC Volatility (z-units) | 0.044 | -0.007 | 0.095 | 1.000 | Practically equivalent |
| f0 mean | -0.049 | -2.020 | 1.880 | 1.000 | Practically equivalent |
| f0 SD | -0.319 | -2.308 | 1.592 | 1.000 | Practically equivalent |
| mean RMS | -0.001 | -0.003 | 0.000 | 1.000 | Practically equivalent |
| RMS SD | 0.001 | -0.001 | 0.004 | 1.000 | Practically equivalent |
| mean spectral bandwidth | -0.023 | -1.969 | 1.998 | 1.000 | Practically equivalent |
| mean spectral centroid | -0.036 | -1.967 | 1.931 | -0.036 | Practically equivalent |
| spectral rolloff (85%) | -0.009 | -1.976 | 1.941 | -0.009 | Practically equivalent |

As a comparison with Experiment 1, we also compared the nPVI between the two stimulus sets of Experiment 2. We did not obtain any significant differences between the two stimulus sets (regular-only mean=37.007; repeating-only mean=40.109, t = -1.488, df = 30.17, p = 0.147).

Experiment 1

Experiment 1 compared preferences for fully rhythmic and arrhythmic stimuli as well as silence, in male and female budgerigars. Here we show additional model results for the model cumdur.sqrt~sound*Sex + (1|subject) + (0+sound.fully_rhythmic|subject) + (0+sound.silence|subject) (cumdur.sqrt = cumulated listening time, square-root transformed to meet model assumptions).

Table S5: 95%-confidence intervals for the full model of experiment 1 (note that our response variable was square root-transformed)

| term | orig | 2.5 CI | 97.5 CI |
| --- | --- | --- | --- |
| Intercept | 71.287 | 47.088 | 97.030 |
| fully rhythmic | 18.462 | -1.810 | 38.109 |
| silence | -22.615 | -47.970 | 2.049 |
| male | -15.979 | -51.648 | 24.481 |
| fully rhythmic:male | -7.028 | -35.777 | 22.720 |
| silence:male | 40.315 | -0.576 | 77.019 |

Table S6: Estimated marginal means for experiment 1, back-transformed to seconds, rounded to the nearest integer.

| sound | Sex | estimated marginal mean | SE | 2.5 CI | 97.5 CI |
| --- | --- | --- | --- | --- | --- |
| arrhythmic | F | 5082 | 1790 | 1925 | 9741 |
| fully rhythmic | F | 8055 | 2500 | 3567 | 14346 |
| silence | F | 2369 | 1640 | 170 | 7104 |
| arrhythmic | M | 3059 | 1500 | 661 | 7210 |
| fully rhythmic | M | 4454 | 2010 | 1177 | 9836 |
| silence | M | 5330 | 2660 | 1193 | 12427 |

Model stability was assessed by using a leave-one-subject-out (LOSO, jackknife) sensitivity analysis for each random effect. For each level, the model was refitted after excluding a level of the random effect (subject), and estimates were compared to the original model. Stability was then assessed by dividing the range of estimates (max-min) by the 95% profile likelihood confidence intervals of the effects of the original models (“stability index”, SI). This measured how much estimates shifted relative to the model’s uncertainty. SI>0.5 were considered possibly unstable and inspected more closely (see Table S7:). This was the case for the effects of Sex, the interaction Sex:stimulus condition, and the random intercept. We subsequently plotted the LOSO estimates against the CI of the original model and inspected whether any resulting estimate would leave the confidence interval or crossed zero (making the fixed effect non-significant, see Figures S3, S4, S5). This was not the case. To measure the influence of single subjects, we calculated the distance between the original estimate and the estimate produced when a specific subject was removed, standardized by the CI width. We used a threshold value of >0.5 as indicating substantial influence; a value of >1 would be critical, as it would mean one subject moves the estimate further than the CI width. Since none of the subjects exceeded the 0.5 threshold, we considered the model reasonably stable.

Table S7: Model stability results for experiment 1 (R code generated with AI assistance). Model stability was assessed using a leave-one-subject-out (LOSO, jackknife) sensitivity analysis for each random effect. For each level, the model was refitted after excluding a level of the random effect (subject), and estimates were compared to the original model. Stability was then assessed by dividing the range of estimates (max-min) by the 95% profile likelihood confidence intervals of the effects of the original models (stability index, SI). This measured how much estimates shifted relative to the model’s uncertainty. SI>0.5 were considered possibly unstable and inspected more closely.

| term | original estimate | min estimate | max estimate | Width of 95% profile CI | SI |
| --- | --- | --- | --- | --- | --- |
| SexM | -15.979 | -27.527 | -5.318 | 26.828 | 0.828 |
| soundsilence:SexM | 40.315 | 26.428 | 52.533 | 39.523 | 0.660 |
| (Intercept) | 71.287 | 64.602 | 82.834 | 27.737 | 0.657 |
| soundfully_rhythmic | 18.462 | 12.225 | 25.470 | 31.832 | 0.416 |
| soundfully_rhythmic:SexM | -7.028 | -14.037 | -0.469 | 48.526 | 0.280 |
| soundsilence | -22.615 | -27.419 | -14.800 | 48.779 | 0.259 |
| sd__(Intercept) | 29.656 | 18.823 | 32.034 | 53.143 | 0.249 |
| sd__sound.silence | 29.784 | 21.929 | 35.676 | 58.176 | 0.236 |
| sd__Observation | 14.855 | 5.513 | 18.485 | 78.224 | 0.166 |
| sd__sound.fully_rhythmic | 16.099 | 11.780 | 20.983 | 71.429 | 0.129 |

Figure S2: Model stability of experiment 1 (R code generated with AI assistance). Stability index (SI). Red bars indicate high sensitivity to single-subject exclusion (SI>0.5). Effects beginning with “sd__” refer to standard deviations of random slopes/intercept.

Figure S3: Model stability of experiment 1 (R code generated with AI assistance). LOSO sensitivity analysis of the effect of soundarrhythmic:SexF (Intercept). Red points indicate estimates when excluding that subject. Blue shaded area indicates the 95% CI of the original model.

Figure S4: Model stability of experiment 1 (R code generated with AI assistance). LOSO sensitivity analysis of the effect of SexM. Red points indicate estimates when excluding that subject. Blue shaded area indicates the 95% CI of the original model.

Figure S5: Model stability of experiment 1 (R code generated with AI assistance). LOSO sensitivity analysis of the effect of soundsilence:SexM. Red points indicate estimates when excluding that subject. Blue shaded area indicates the 95% CI of the original model.

Exploratory analysis separately for each Sex

As we suspected that our study was underpowered to render female preferences significant, we conducted post-hoc tests using linear mixed models for each sex separately (including "subject" as a random intercept and random slopes across stimulus conditions). Note that this approach should be regarded as exploratory. Full-null model comparisons revealed significant effects of condition in females (χ^2^ = 8.952, df = 2, p = 0.01, R2m = 0.198, R2c = 0.717) but not in males (χ^2^ = 2.306, df = 2, p = 0.316, R2m = 0.032, R2c = 0.956). FDR-corrected pairwise contrasts for female data (R package "emmeans", version 1.8.4-1, ^9^) revealed that females significantly preferred fully rhythmic stimuli (estimated marginal mean = 8046) over silence (estimated marginal mean = 2372, t-ratio = 3.546, p_FDR_ = 0.02), while the other contrasts remained non-significant (arrhythmic-fully rhythmic: t-ratio = -1.594, p_FDR_ = 0.156; arrhythmic-silence: t-ratio = 2.052, p_FDR_ = 0.121; arrhythmic estimated marginal mean = 5084).

Bayesian comparison of results from Hoeschele & Bowling, 2016 ^10^, and Experiment 1

Both Hoeschele & Bowling, 2016 ^10^, and the current experiment 1 suggested a latent preference for rhythmic stimuli in female budgerigars. To estimate evidence for this latent preference given both studies we analyzed listening behavior using a hierarchical Bayesian Dirichlet regression, modeling the proportion of total listening time each bird allocated to each stimulus category (R code generated with AI assistance). Model formulas were cbind(prop_rhy, prop_arr) ~ Sex + (1 | subject) + (0 + Sex | subject) for Hoeschele & Bowling, 2016, and cbind(prop_rhy, prop_arr, prop_sil) ~ Sex + (1 | subject) + (0 + Sex | subject) for experiment 1. Here we show additional model results.

Table S8. Summary output of Dirichlet model of data from ^10^ (N=16).

| Parameter | Estimate | Est.Error | l-95% CI | u-95% CI | Rhat | Bulk_ESS | Tail_ESS |
| --- | --- | --- | --- | --- | --- | --- | --- |
| sd(muproparr_Intercept) | 1.68 | 1.04 | 0.07 | 3.97 | 1.00 | 1428 | 2777 |
| sd(muproparr_SexF) | 1.33 | 0.94 | 0.06 | 3.55 | 1.00 | 1958 | 3699 |
| sd(muproparr_SexM) | 2.77 | 1.46 | 0.24 | 5.99 | 1.00 | 1481 | 2110 |
| cor(muproparr_SexF,muproparr_SexM) | 0.00 | 0.58 | -0.95 | 0.95 | 1.00 | 2257 | 4400 |
| muproparr_Intercept | -1.18 | 0.97 | -3.19 | 0.66 | 1.00 | 5446 | 4645 |
| muproparr_SexM | 1.44 | 1.64 | -1.68 | 4.84 | 1.00 | 5237 | 5455 |
| phi | 15.33 | 22.55 | 1.07 | 75.52 | 1.01 | 872 | 2016 |

Table S9: Summary output of the Dirichlet model of data from experiment 1 (N=13).

| Parameter | Estimate | Est.Error | l-95% CI | u-95% CI | Rhat | Bulk_ESS | Tail_ESS |
| --- | --- | --- | --- | --- | --- | --- | --- |
| sd(muproparr_Intercept) | 0.39 | 0.27 | 0.02 | 1.02 | 1.00 | 1990 | 3864 |
| sd(muproparr_SexF) | 0.48 | 0.36 | 0.02 | 1.36 | 1.00 | 2793 | 3446 |
| sd(muproparr_SexM) | 0.59 | 0.47 | 0.02 | 1.76 | 1.00 | 2940 | 3732 |
| sd(mupropsil_Intercept) | 1.04 | 0.58 | 0.08 | 2.26 | 1.00 | 1896 | 3027 |
| sd(mupropsil_SexF) | 1.25 | 0.82 | 0.08 | 3.15 | 1.00 | 2159 | 3688 |
| sd(mupropsil_SexM) | 1.10 | 0.79 | 0.05 | 3.04 | 1.00 | 2483 | 3491 |
| cor(muproparr_SexF,muproparr_SexM) | -0.01 | 0.57 | -0.95 | 0.95 | 1.00 | 4767 | 5606 |
| cor(mupropsil_SexF,mupropsil_SexM) | -0.01 | 0.58 | -0.95 | 0.95 | 1.00 | 3880 | 5259 |
| muproparr_Intercept | -0.40 | 0.33 | -1.08 | 0.26 | 1.00 | 6194 | 5170 |
| mupropsil_Intercept | -1.77 | 0.77 | -3.33 | -0.26 | 1.00 | 4146 | 4477 |
| muproparr_SexM | 0.15 | 0.54 | -0.91 | 1.25 | 1.00 | 5454 | 4336 |
| mupropsil_SexM | 1.73 | 1.11 | -0.51 | 3.98 | 1.00 | 4299 | 4289 |
| phi | 46.27 | 43.69 | 9.00 | 161.06 | 1.00 | 814 | 1776 |

Experiment 2

Experiment 2 compared preferences for regular-only and repeating-only stimuli as well as silence, in female budgerigars. Stability analysis was done as in experiment 1. We observed an SI>0.5 for the random intercept and the random slope sound:repeating_only. Influence analysis (distance between the original estimate and the estimate produced when removing a subject, standardized by the original CI width) revealed a substantial influence of one bird (“Melanzani”, 0.512 for the random intercept, 0.936 for the random slope; listening time in repeating-only more than two SD below the group mean). When excluding this bird, subject-level SD dropped to zero. However, we retained the random intercept in the subsequent simplified model (excluding “Melanzani”) to respect the hierarchical structure of the experimental design. Here we show additional model results for the model cumdur.sqrt~sound + (1|subject)+(0+sound.repeating_only|subject)+(0+sound.silence|subject) (cumdur.sqrt = cumulated listening time, square-root transformed to meet model assumptions). We also show additional results for the simplified model excluding bird “Melanzani” (cumdur.sqrt~sound + (1|subject)).

Table S10: 95%-confidence intervals for the initial full model of experiment 2 (including the bird “Melanzani”). Note that in the model the response variable was square-root-transformed.

| term | orig | 2.5 CI | 97.5 CI |
| --- | --- | --- | --- |
| Intercept | 86.136 | 76.748 | 96.334 |
| repeating only | -4.028 | -22.974 | 13.857 |
| silence | -30.331 | -42.966 | -17.072 |

Table S11: Estimated marginal means of durations for the initial full model of experiment 2, back-transformed to seconds, rounded to integers.

| sound | estimated marginal mean | SE | 2.5 CI | 97.5 CI |
| --- | --- | --- | --- | --- |
| regular only | 7419 | 879 | 5797 | 9242 |
| repeating only | 6741 | 1590 | 3978 | 10230 |
| Silent | 3114 | 774 | 1782 | 4816 |

Figure S6: Estimated marginal means of cumulative listening duration (responses transformed back to seconds) for female budgerigars, including the bird “Melanzani”, in the three-place preference paradigm (silence, repeating-only, and regular-only stimuli). Points indicate model-based estimates; error bars represent 95% confidence intervals.

Figure S7: Model stability of experiment 2 (R code generated with AI assistance). Stability index (SI). Red bars indicate high sensitivity to single-subject exclusion (SI>0.5). Effects beginning with “sd__” refer to standard deviations of random slopes/intercept.

Figure S8: Model stability of experiment 2 (R code generated with AI assistance). LOSO sensitivity analysis of the random slope sound.repeating_only. Red points indicate the standard deviation of this effect when excluding that subject. Blue shaded area indicates the 95% CI of the original model. Note the strong deviation of the model estimate when subject “Melanzani” is excluded.

Figure S9: Model stability of experiment 2 (R code generated with AI assistance). LOSO sensitivity analysis of the random intercept. Red points indicate the standard deviation of this effect when excluding that subject. Blue shaded area indicates the 95% CI of the original model. Note that the effect’s standard deviation drops to zero when subject “Melanzani” is excluded. This indicates that “Melanzani” is the only subject with a baseline performance different enough from the group mean to justify a random intercept. However, we retained the random intercept in the subsequent simplified model (excluding “Melanzani”) to respect the hierarchical structure of the experimental design.

Table S12: Model stability results for experiment 2 (R code generated with AI assistance).

| term | original estimate | min estimate | max estimate | Width of 95% profile CI | SI |
| --- | --- | --- | --- | --- | --- |
| sd__sound.repeating_only | 28.633 | 9.454 | 31.576 | 20.499 | 1.079 |
| sd__(Intercept) | 13.406 | 0.000 | 15.105 | 26.191 | 0.577 |
| sd__Observation | 11.508 | 9.691 | 19.480 | 30.904 | 0.317 |
| (Intercept) | 86.136 | 83.309 | 89.653 | 25.615 | 0.248 |
| soundrepeating_only | -4.028 | -8.736 | 0.472 | 48.602 | 0.189 |
| soundsilence | -30.331 | -32.592 | -27.926 | 31.551 | 0.148 |
| sd__sound.silence | 16.274 | 13.636 | 18.463 | 38.682 | 0.125 |

Table S13: 95%-confidence intervals for the simplified model of experiment 2 (excluding the bird “Melanzani”). Note that in the model the response variable was square-root-transformed.

| term | orig | 2.5 CI | 97.5 CI |
| --- | --- | --- | --- |
| Intercept | 86.684 | 74.439 | 101.165 |
| repeating only | 0.472 | -18.716 | 18.668 |
| silence | -31.093 | -51.140 | -12.009 |

Table S14: Estimated marginal means of durations for the simplified model of experiment 2, back-transformed to seconds, rounded to integers.

| sound | estimated marginal mean | SE | 2.5 CI | 97.5 CI |
| --- | --- | --- | --- | --- |
| regular only | 7514 | 1190 | 5301 | 10112 |
| repeating only | 7596 | 1190 | 5370 | 10208 |
| Silent | 3090 | 761 | 1740 | 4826 |

Experiment 3

In experiment 3, we tested humans in a highly similar setup as budgerigars (three-place preference paradigm, each session comprising the choice between two stimulus conditions and silence), using four stimulus sets: arrhythmic, repeating-only, regular-only, fully rhythmic. Starting from the full initial model cumdur ~ sound * max_instrument_years + sound * Gender + (1|subject) + (0+sound.arrhythmic|subject) + (0+sound.repeating_only|subject) + (0+sound.regular_only|subject) + (0+sound.fully_rhythmic|subject), we also report additional results for the models simplified for stability reasons.

Stability (LOSO) analysis revealed that the initial full model led to instability across a variety of effects, suggesting over-parameterisation of the model. We therefore tested a simplified model devoid of random slopes against the initial model in a likelihood ratio test, resulting in a non-significant result (χ^2^ =0.044, df=4, p>0.999, R2m=0.504, R2c=0.619). Since the models were statistically indistinguishable, we continued the analysis with the simplified model. Likelihood ratio tests of the interactions revealed neither a significant interaction of sound:gender (F(4,152)= 2.404, p=0.052) nor of sound:max_instrument_years ( F(4,152)=0. 193, p=0.942).

Table S15: 95%-confidence intervals for the initial full model of experiment 3

| term | orig | 2.5 CI | 97.5 CI |
| --- | --- | --- | --- |
| (Intercept) | 95.804 | 53.531 | 138.077 |
| soundarrhythmic | 171.308 | 118.197 | 224.420 |
| soundrepeating-only | 155.470 | 102.359 | 208.581 |
| soundregular-only | 208.505 | 154.831 | 262.179 |
| soundfully rhythmic | 236.909 | 183.801 | 290.017 |
| max_instrument_years | -2.515 | -7.381 | 2.350 |
| Genderm | 19.461 | -41.839 | 80.762 |
| soundarrhythmic:max_instrument_years | -0.725 | -6.838 | 5.388 |
| soundrepeating-only:max_instrument_years | 1.216 | -4.897 | 7.329 |
| soundregular-only:max_instrument_years | 1.464 | -4.714 | 7.642 |
| soundfully rhythmic:max_instrument_years | 1.312 | -4.800 | 7.425 |
| soundarrhythmic:Genderm | -101.320 | -178.337 | -24.303 |
| soundrepeating-only:Genderm | -43.451 | -120.468 | 33.565 |
| soundregular-only:Genderm | -80.692 | -158.525 | -2.860 |
| soundfully rhythmic:Genderm | -98.501 | -175.513 | -21.490 |

Figure S10: Model stability of the initial model of experiment 3 (R code generated with AI assistance). Stability index (SI). Red bars indicate high sensitivity to single-subject exclusion (SI>0.5). Effects beginning with “sd__” refer to standard deviations of random slopes/intercept.

Table S16: 95%-confidence intervals for the simplified model (without random slopes) of experiment 3

| term | orig | 2.5 CI | 97.5 CI |
| --- | --- | --- | --- |
| (Intercept) | 95.803 | 53.409 | 138.199 |
| soundarrhythmic | 171.308 | 118.034 | 224.582 |
| soundrepeating_only | 155.470 | 102.196 | 208.744 |
| soundregular_only | 208.505 | 155.231 | 261.779 |
| soundfully_rhythmic | 236.909 | 183.635 | 290.183 |
| max_instrument_years | -2.515 | -7.395 | 2.364 |
| Genderm | 19.461 | -42.015 | 80.938 |
| soundarrhythmic:max_instrument_years | -0.725 | -6.857 | 5.407 |
| soundregular_only:max_instrument_years | 1.464 | -4.668 | 7.596 |
| soundfully_rhythmic:max_instrument_years | 1.312 | -4.819 | 7.444 |
| soundarrhythmic:Genderm | -101.320 | -6.857 | 5.407 |
| soundrepeating_only:Genderm | -43.451 | -120.704 | 33.801 |
| soundregular_only:Genderm | -80.693 | -157.945 | -3.440 |
| soundfully_rhythmic:Genderm | -98.501 | -175.754 | -21.249 |

Figure S11: Model stability of the simplified model of experiment 3 (no random slopes) (R code generated with AI assistance). Stability index (SI). Red bars indicate high sensitivity to single-subject exclusion (SI>0.5). Effects beginning with “sd__” refer to standard deviations of random slopes/intercept.

Figure S12: Model stability of the simplified model of experiment 3 (no random slopes) (R code generated with AI assistance). LOSO sensitivity analysis of soundregular_only:Genderm. Red points indicate the standard deviation of this effect when excluding that subject. Blue shaded area indicates the 95% CI of the original model.

Figure S13: Model stability of the simplified model of experiment 3 (no random slopes) (R code generated with AI assistance). LOSO sensitivity analysis of soundarrhythmic. Red points indicate the standard deviation of this effect when excluding that subject. Blue shaded area indicates the 95% CI of the original model.

Figure S14: Model stability of the simplified model of experiment 3 (no random slopes) (R code generated with AI assistance). LOSO sensitivity analysis of soundsilence:Genderf (Intercept). Red points indicate the standard deviation of this effect when excluding that subject. Blue shaded area indicates the 95% CI of the original model.

Figure S15: Model stability of the simplified model of experiment 3 (no random slopes)(R code generated with AI assistance). LOSO sensitivity analysis of soundregular_only. Red points indicate the standard deviation of this effect when excluding that subject. Blue shaded area indicates the 95% CI of the original model.

Figure S16: Model stability of the simplified model of experiment 3 (no random slopes) (R code generated with AI assistance). LOSO sensitivity analysis of soundarrhythmic:Genderm. Red points indicate the standard deviation of this effect when excluding that subject. Blue shaded area indicates the 95% CI of the original model.

Table S17: 95%-confidence intervals for the final model of experiment 3 (containing only main effects of sound, gender, max_instrument years, and a random intercept for subjects).

| term | orig | 2.5 CI | 97.5 CI |
| --- | --- | --- | --- |
| (Intercept) | 107.384 | 73.878 | 140.889 |
| soundarrhythmic | 143.134 | 109.569 | 176.698 |
| soundrepeating_only | 152.186 | 118.621 | 185.750 |
| soundregular_only | 197.830 | 164.266 | 231.395 |
| soundfully_rhythmic | 221.143 | 187.578 | 254.707 |
| Genderm | -45.332 | -83.399 | -7.264 |
| max_instrument_years | -1.862 | -4.883 | 1.160 |

Table S18: Estimated marginal means for the final model of experiment 3 (containing only main effects of sound, gender, max_instrument years, and a random intercept for subjects).

| sound | emmean | SE | lower.CL | upper.CL |
| --- | --- | --- | --- | --- |
| silence | 74 | 14.6 | 45 | 103 |
| arrhythmic | 217 | 14.6 | 188 | 246 |
| repeating_only | 226 | 14.6 | 197 | 255 |
| regular_only | 272 | 14.6 | 243 | 301 |
| fully_rhythmic | 295 | 14.6 | 266 | 324 |

Table S19: Results of FDR-corrected post-hoc tests for the final model of experiment 3 (containing only main effects of sound, gender, max_instrument years, and a random intercept for subjects)

| contrast | estimate | SE | df | t.ratio | p.value |
| --- | --- | --- | --- | --- | --- |
| silence - arrhythmic | -143.13 | 17.2 | 148 | -8.300 | <0.001 |
| silence - repeating_only | -152.19 | 17.2 | 148 | -8.825 | <0.001 |
| silence - regular_only | -197.83 | 17.2 | 148 | -11.471 | <0.001 |
| silence - fully_rhythmic | -221.14 | 17.2 | 148 | -12.823 | <0.001 |
| arrhythmic - repeating_only | -9.05 | 17.2 | 148 | -0.525 | 0.600 |
| arrhythmic - regular_only | -54.70 | 17.2 | 148 | -3.172 | 0.002 |
| arrhythmic - fully_rhythmic | -78.01 | 17.2 | 148 | -4.523 | <0.001 |
| repeating_only - regular_only | 45.64 | 17.2 | 148 | 2.647 | 0.011 |
| repeating_only - fully_rhythmic | -68.96 | 17.2 | 148 | -3.999 | <0.001 |
| regular_only - fully_rhythmic | -23.31 | 17.2 | 148 | -1.352 | 0.198 |
| female - male | 45.3 | 19.7 | 35 | 2.298 | 0.028 |

Rating of stimuli

After the main experiment participants were asked to re-listen to random sample stimuli from each stimulus set and rate them on 7-point Likert scales (from “not at all” to “very”) for three questions: (1) “How beautiful did you find the audio file?”; (2) “How interesting did you find the audio file?”; and (3) “How meaningful did you find the audio file?”. Here we report the results of analyzing the ratings.

We analyzed the rating data using Friedman tests (due to data being ordinal, R package "stats", version 4.2.1, function "friedman.test", ^11^) and found that responses differed significantly across conditions for all three questions ("Beautiful": χ^2^(3)=56.622, p<0.001; "Interesting": χ^2^(3)=25.293, p<0.001;"Makes sense": χ^2^(3)=37.685, p<0.001).

We then conducted paired Wilcoxon tests (FDR-corrected, R package "exactRankTests", version 0.8-35, ^12^ as post-hoc tests for the data of each question (see Table S20). The results reflected the pattern observed in the place preference paradigm for all three questions, that is, a significant preference for fully rhythmic and a slightly less pronounced but significant preference for regular-only over repeating-only and arrhythmic. Additionally, fully rhythmic stimuli were significantly preferred over regular only stimuli regarding ratings for beauty and meaningfulness.

Table S20: Results of Wilcoxon post-hoc tests (FDR corrected, V and WT are the rank sums, N is the sample size without ties).

| Question | comparison | Median 1 | Median 2 | N | V | WT | z | p |
| --- | --- | --- | --- | --- | --- | --- | --- | --- |
| "Beautiful" | arrhythmic - regular only | arrhythmic: 3 | regular only: 4 | 31 | 65 | 431 | 3.586 | <0.001 |
|  | arrhythmic - repeating only | arrhythmic: 3 | repeating only: 2 | 29 | 188.5 | 246.5 | 0.627 | 0.560 |
|  | arrhythmic - fully rhythmic | arrhythmic: 3 | fully rhythmic: 5 | 36 | 28 | 638 | 4.792 | <0.001 |
|  | regular only - repeating only | regular only: 4 | repeating only: 2 | 31 | 409 | 409 | 3.155 | 0.001 |
|  | regular only - fully rhythmic | regular only: 4 | fully rhythmic: 5 | 32 | 47 | 481 | 4.058 | <0.001 |
|  | repeating only - fully rhythmic | repeating only: 2 | fully rhythmic: 5 | 34 | 28.5 | 566.5 | 4.599 | <0.001 |
| "Interesting" | arrhythmic - regular only | arrhythmic: 3.5 | regular only: 5 | 28 | 58 | 348 | 3.302 | <0.001 |
|  | arrhythmic - repeating only | arrhythmic: 3.5 | repeating only: 3 | 36 | 348.5 | 348.5 | 0.244 | 0.810 |
|  | arrhythmic - fully rhythmic | arrhythmic: 3.5 | fully rhythmic: 5 | 33 | 53 | 508 | 4.065 | <0.001 |
|  | regular only - repeating only | regular only: 5 | repeating only: 3 | 29 | 349 | 349 | 2.843 | 0.005 |
|  | regular only - fully rhythmic | regular only: 5 | fully rhythmic: 5 | 29 | 150 | 285 | 1.460 | 0.167 |
|  | repeating only - fully rhythmic | repeating only: 3 | fully rhythmic: 5 | 36 | 81.5 | 584.5 | 3.951 | <0.001 |
| "Meaningful" | arrhythmic - regular only | arrhythmic: 2 | regular only: 3 | 27 | 45 | 333 | 3.4596 | <0.001 |
|  | arrhythmic - repeating only | arrhythmic: 2 | repeating only: 2 | 23 | 104.5 | 171.5 | 1.019 | 0.337 |
|  | arrhythmic - fully rhythmic | arrhythmic: 2 | fully rhythmic: 4 | 32 | 22.5 | 505.5 | 4.516 | <0.001 |
|  | regular only - repeating only | regular only: 3 | repeating only: 2 | 25 | 273.5 | 273.5 | 2.987 | 0.003 |
|  | regular only - fully rhythmic | regular only: 3 | fully rhythmic: 4 | 26 | 87.5 | 263.5 | 2.235 | 0.026 |
|  | repeating only - fully rhythmic | repeating only: 2 | fully rhythmic: 4 | 28 | 29 | 377 | 3.962 | <0.001 |

Figure S17. Rating sheet used to rate sample stimuli after the place-preference experiment, using 7-point Likert scales. Note: The letters “A”, “P”, “R”, and “M” represented our internal labelling and were chosen to not give any information about the purpose of the experiment away. Since none of the participants reported in the post-experimental questionnaire that they had guessed the labels, we do not expect that they caused any participant biases.

Effect of participants guessing the study purpose

Our post-experimental questionnaire revealed that 20 out of the 38 participants guessed that the study was about “preference”, “rhythm” or “patterns” (with no gender imbalance between the guessers and non-guessers: χ^2^(1)=1.184, p=0.277).We therefore tested whether this guessing effect influenced the results. Using a likelihood ratio test, we found a significant main effect of guessing (χ^2^(1)=8.342, p=0.004), with guessers having a significantly higher estimated marginal mean (240) than non-guessers (196, FDR-corrected post-hoc comparison: SE=15.2, df=41.3, t.ratio=-2.931, p=0.006). However, we did not observe an interaction with sound (χ^2^(4)=6.256, p=0.181), thus, guessers listened more to the stimuli overall, but the resulting pattern stayed the same.

Figure S18: Effect of participants guessing the study purpose, based on the post-experimental questionnaire (free text answers). We did not find an interaction with the stimulus condition (thus not affecting the overall pattern), but a main effect with participants guessing the study involved “rhythm”, “preferences” or “patterns” listening slightly longer to the stimuli. We deliberately used these keywords as a liberal criterion for determining a correct guessing to make sure our analysis was valid.

Additional analysis of the subset of human data comprising only sessions comparing repeating-only, regular-only, and silence

To enhance the comparability between the results of female budgerigars from experiment 2 where budgerigars could choose between silence, regular-only and repeating-only stimuli, and those of humans from experiment 3 (for which we included additional comparisons with arrhythmic and fully rhythmic stimuli), we analyzed a subset of the human data only comprising sessions where participants could choose between these three stimulus conditions (thus, comprising sessions comparing repeating-only, regular-only, and silence). With this matched-design validity analysis we aimed to assess whether either of the two stimulus sets was preferred significantly more often under these circumstances. Because the main analysis had revealed no effect of musical training, we used a simplified model with fixed effects for stimulus condition and gender and a random intercept for subject (model formula: cumdur ~ sound + Gender + (1|subject)). The full-null model comparison was significant (χ^2^ = 84.440, df = 4, p <= 0.001, R2m = 0.526, R2c = 0.530) with no variance explained by the random intercept, indicating high consistency among participants. We found a significant effect of stimulus condition (F(2,72.471)= 59.897, p < 0.001, R2m = 0.010, R2c = 0.010) but not of gender (F(2,34.136)= 1.270, p = 0.294). LOSO analysis revealed that the model was reasonably stable. FDR-corrected post-hoc pairwise comparisons revealed significant preferences between all stimulus conditions whereby regular-only stimuli were preferred over repeating-only stimuli, unlike for the budgerigars (see Table S21).

Table S21: FDR-corrected post-hoc pairwise comparisons for the matched-design validation analysis (for comparability with Exp. 2) using only the subset of human data comprising sessions comparing repeating-only, regular-only and silence. Due to the non-significant effect of gender, results are averaged over the levels of gender.

| contrast | estimate | SE | df | t.ratio | p.value |
| --- | --- | --- | --- | --- | --- |
| regular_only - repeating_only | 16.3 | 7.77 | 70.9 | 2.100 | 0.039 |
| regular_only - silence | 79.3 | 7.67 | 72.2 | 10.333 | <0.001 |
| repeating_only - silence | 63.0 | 7.67 | 72.2 | 8.205 | <0.001 |

Participant Demographics

Table S21: Self-reported demographic data of tested cohort. Demographic characteristics including race, ethnicity, and socioeconomic income were not collected in accordance with regional institutional review board guidelines and data privacy regulations (GDPR) regarding sensitive personal data in the European Union. Geographic distribution and linguistic background were recorded to provide alternative cohort context. Full questionnaire data can be found under *https://osf.io/mj4kp/overview.*

| **Demographic Variable** | **Subvariables** | **Value** |
| --- | --- | --- |
| Age | Mean $\pm$ SD [Range] | 23 $\pm$ 6.4 [18-43] |
| Gender | female | N=29 |
|  | male | N=9 |
|  | non-binary/other | N=0 |
| Geographic distribution | Living in Austria | N=38 (100%) |
|  | Lived only in Austria | N=11 (28.9%) |
|  | Lived in 2 countries in total | N=18 (47.4%) |
|  | Lived in ≥ 3 countries in total | N=9 (23.7%) |
|  | Years living in Austria, Mean $\pm$ SD [Range] | 13.3 $\pm$ 12.4 [0.5-40.5] |
|  | Years living within EU (outside Austria), Mean $\pm$ SD [Range] | 12.7 $\pm$ 10.1 [0.5-37] |
|  | Years living outside EU, Mean $\pm$ SD [Range] | 12.6 $\pm$ 9.6 [0.17-23] |
| Linguistic background | Monolingual [German] | N=3 (7.9%) |
|  | Multilingual | N=35 (92.1%) |

Post-experimental questionnaire

Figure S19: post-experimental questionnaire used to assess participant data, including questions about musical experience (we used the maximum number of years across instruments reported in question 4 as our measure for “musical training”).

Figure S20: Diagram of human testing setup (Exp. 3)

References

1.	Cannam, C., Landone, C. & Sandler, M. Sonic visualiser: An open source application for viewing, analysing, and annotating music audio files. in *MM’10 - Proceedings of the ACM Multimedia 2010 International Conference* 1467–1468 (2010). doi:10.1145/1873951.1874248.

2.	Okanoya, K. & Dooling, R. J. *Hearing in Passerine and Psittacine Birds: A Comparative Study of Absolute and Masked Auditory Thresholds*. *Journal of Comparative Psychology* vol. 101 (1987).

3.	Dooling, R. J. & Saunders, J. C. Hearing in the parakeet (Melopsittacus undulatus): absolute thresholds, critical ratios, frequency difference limens, and vocalizations. *J. Comp. Physiol. Psychol.* **88**, (1975).

4.	Dent, M. L., Dooling, R. J. & Pierce, A. S. *Frequency Discrimination in Budgerigars (Melopsittacus Undulatus): Effects of Tone Duration and Tonal Context*. (2000).

5.	Dooling, R. J., Lohr, B. & Dent, M. L. Hearing in birds and reptiles. in *Comparative hearing: birds and reptiles* 308–359 (Springer).

6.	Bregman, M. R., Patel, A. D. & Gentner, T. Q. Songbirds use spectral shape, not pitch, for sound pattern recognition. *Proc. Natl. Acad. Sci. U. S. A.* **113**, 1666–1671 (2016).

7.	Henry, K. S., Amburgey, K. N., Abrams, K. S., Idrobo, F. & Carney, L. H. Formant-frequency discrimination of synthesized vowels in budgerigars ( Melopsittacus undulatus ) and humans . *J. Acoust. Soc. Am.* **142**, 2073–2083 (2017).

8.	Grabe, E. & Low, E. L. Durational Variability in Speech and the Rhythm Class Hypothesis. *Papers in Laboratory Phonology* 515–46 (2002).

9.	Lenth, R. emmeans: Estimated Marginal Means, aka Least-Squares Means. (2023).

10.	Hoeschele, M. & Bowling, D. L. Sex differences in rhythmic preferences in the Budgerigar (Melopsittacus undulatus): A comparative study with humans. *Front. Psychol.* **7**, (2016).

11.	R Core Team. R: A Language and Environment for Statistical Computing (Version 4.4.0). *R Foundation for Statistical Computing, Vienna, Austria.* https://www.R-project.org (2025).

12.	Hothorn, T., Hornik, K. & Hothorn, M. T. Package ‘exactRankTests’. *Version 0.8-35* (2022).
